# Impact of Tellurite on the Metabolism of *Paenibacillus pabuli* AL109b With Flagellin Production Explaining High Reduction Capacity

**DOI:** 10.3389/fmicb.2021.718963

**Published:** 2021-09-07

**Authors:** Pedro Farias, Romeu Francisco, Lorrie Maccario, Jakob Herschend, Ana Paula Piedade, Søren Sørensen, Paula V. Morais

**Affiliations:** ^1^Department of Life Sciences, CEMMPRE, University of Coimbra, Coimbra, Portugal; ^2^Section of Microbiology, Department of Biology, University of Copenhagen, Copenhagen, Denmark; ^3^CEMMPRE, Department Mechanical Engineering, University of Coimbra, Coimbra, Portugal

**Keywords:** *Paenibacillus* sp., genome, proteome, flagellin, tellurite

## Abstract

Tellurium (Te) is a metalloid with scarce and scattered abundance but with an increased interest in human activity for its uses in emerging technologies. As is seen for other metals and metalloids, the result of mining activity and improper disposal of high-tech devices will lead to niches with increased abundance of Te. This metalloid will be more available to bacteria and represent an increasing selective pressure. This environmental problem may constitute an opportunity to search for microorganisms with genetic and molecular mechanisms of microbial resistance to Te toxic anions. Organisms from Te-contaminated niches could provide tools for Te remediation and fabrication of Te-containing structures with added value. The objective of this study was to determine the ability of a high metal-resistant *Paenibacillus pabuli* strain ALJ109b, isolated from high metal content mining residues, to reduce tellurite ion, and to evaluate the formation of metallic tellurium by cellular reduction, isolate the protein responsible, and determine the metabolic response to tellurite during growth. *P. pabuli* ALJ109b demonstrated to be resistant to Te (IV) at concentrations higher than reported for its genus. It can efficiently remove soluble Te (IV) from solution, over 20% in 8 h of growth, and reduce it to elemental Te, forming monodisperse nanostructures, verified by scattering electron microscopy. Cultivation of *P. pabuli* ALJ109b in the presence of Te (IV) affected the general protein expression pattern, and hence the metabolism, as demonstrated by high-throughput proteomic analysis. The Te (IV)-induced metabolic shift is characterized by an activation of ROS response. Flagellin from *P. pabuli* ALJ109b demonstrates high Te (0) forming activity in neutral to basic conditions in a range of temperatures from 20°C to 37°C. In conclusion, the first metabolic characterization of a strain of *P. pabuli* response to Te (IV) reveals a highly resistant strain with a unique Te (IV) proteomic response. This strain, and its flagellin, display, all the features of potential tools for Te nanoparticle production.

## Introduction

The study of the Te–bacteria interaction has been mainly focused on resistance to soluble Te ions, particularly the reduction of Te (IV) and Te (VI) to Te (0). This characteristic resulted in a growing interest in isolation and characterization of new organisms with potential in Te ion reduction from a large number of different environments, such as sea sediments (Csotonyi et al., 2006; Ollivier et al., 2008), mine tailings (Maltman et al., 2015), and fouled waters (Chien and Han, 2009). These environments can provide organisms with novel genes and processes to deal with toxic Te (IV), different from those identified in the majority of bacterial strains studied so far, mainly from clinical settings. Tellurite resistance by reduction (TeR) targets the Te oxyanions, and to this date, few mechanisms have been identified as TeR. Among the most well-described genetic clusters involved in tellurium ion resistance are the mechanisms encoded by the gene cluster *terZABCDEF* (Kormutakova et al., 2000), the *tehAB* gene cluster (Lohmeier-Vogel et al., 2004), or the *kilA* operon (Turner et al., 1994, 1995). The relation of these specific Te resistance mechanisms with Te (IV) reduction is in most cases still to be proven. Several works describe mechanisms of Te resistance by unspecific intracellular reduction of Te ions, implicating reducing agents such as nitrate reductases or elements of the respiratory chain (Sabaty et al., 2001; Chasteen et al., 2009; Theisen et al., 2013; Alavi et al., 2014). In most of these cases, TeR is viewed as the main mechanism for Te resistance. Bioreduction of Te occurs when cells interact with soluble and toxic forms of Te (IV) and Te (VI) and convert the oxyanions to an inert and insoluble form. Bioreduction is a relevant biotechnological characteristic to determine, as varies among different organisms; therefore, for new bacterial strains, the reduction efficiency should be determined. The bioreduction to Te can lead to the formation of nanostructures (Baesman et al., 2007; Zare et al., 2012; Presentato et al., 2016; Wang et al., 2018). As verified for other metals, the formation of Te-containing intra-/extra-cellular nanostructures can be monitored by following the bioreduction process. A diversity of microorganisms has shown the capacity to form these nanostructures, such as *Enterobacter cloacae* (Contreras et al., 2018), *Shewanella* sp. (Vaigankar et al., 2018), and *Ochrobactrum* sp. (Zonaro et al., 2017), and extensive work performed on *Rhodobacter capsulatus* (Borghese et al., 2014; Borghese et al., 2017). An increasing interest in understanding the formation of these structures is the result of the growing potential range of applications for bio-produced nanoparticles covering fields such as optical imaging (Plaza et al., 2016) or novel battery technology (Kim et al., 2015). Growing attention has been given to *Paenibacillus* spp. for its potential in biotechnological applications (Jimoh and Lin, 2019; Du et al., 2021). To this date, some studies on the interactions of *Paenibacillus* strains with metals have been produced (Knuutinen et al., 2019; Ogunyemi et al., 2020) but only a few concerning Te (Chien and Han, 2009). Strains of *Paenibacillus* have been characterized for their biochemistry and proteomics and considered of interest in rhizostabilization of cadmium (Kumari and Thakur, 2018) for their high metal resistance, siderophore production, biocontrol activities, and xenobiotic degradation. Additionally, *Paenibacillus* is also known to produce extracellular polysaccharides with high metal ion uptake ability (Prado Acosta et al., 2005). Nowadays, technologies such as differential proteomics give new perspectives in molecular mechanisms of stress response and metal resistance (Moreno and Rojo, 2013; Djoko et al., 2017). Therefore, it can be applied for determining the impact of Te (IV) on microorganism metabolism.

Residues from the Panasqueira mine in the center of Portugal showed to have *Paenibacillus* in their microbial community, which were isolated in the presence of Te. Considering their metabolic versatility, we hypothesized that the genomic and metabolic characterization of the strain would bring to knowledge new biological strategies to cope with Te, able to be explored biotechnologically.

In this work, we aimed to study the metabolism of a *Paenibacillus pabuli* strain ALJ109b able to resist and to reduce Te (IV) to elemental Te. The resulting Te structures were characterized and revealed an organized structure at the nanoscale size. The genome and proteome analysis performed to describe the *P. pabuli* ALJ109b response to Te (IV) revealed the diversity of strategies of this strain to cope with the metalloid. *P. pabuli* ALJ109b showed to shift its metabolism to deal with the Te (IV)-induced oxidative stress and is able to resist high Te (IV) concentrations by reducing the metalloid. Moreover, the *P. pabuli* ALJ109b flagellin was identified as part of the TeR process. The protein was cloned in a recombinant system and its ability to reduce Te (IV) demonstrated.

The current study offers new insights on the metabolism activated by *Paenibacillus* strain in the presence of Te (IV) and identifies the mechanisms by which this strain, using flagellin, effectively produces Te nanoparticles. Flagellin demonstrates potential application in Te (IV) decontamination and in the fabrication of Te nanoparticles.

## Results

### Tellurite Resistance and Reduction by *Paenibacillus pabuli* ALJ109b

**FIGURE 1:**
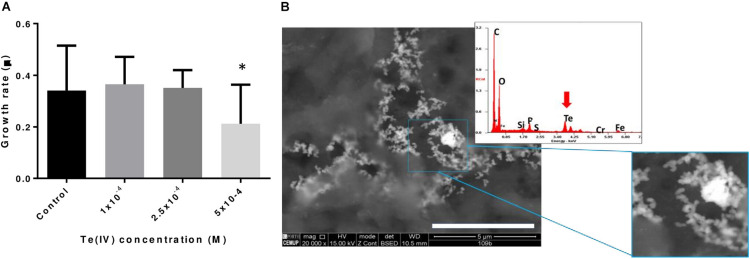
Impact of tellurite in bacterial growth and tellurite reduction. **(A)** Specific growth rates of *Paenibacillus pabuli* ALJ109b at increasing concentrations of Te (IV): control without metal, 1 × 10^–4^ M Te (IV), 2.5 × 10^–4^ M, and 5 × 10^–4^ M. Experiments were conducted in triplicate, and statistical significance indicated ^∗^*p* ≤ 0.05. **(B)** SEM micrographs of Te nanostructures produced with *P. pabuli* ALJ109b cells grown in the presence of 5 × 10^–4^ M Te (IV). A magnification is present for clarification of the nanostructure shape. High-density metal deposits are represented in white. The EDS spectrum was obtained from reads in an electrodense area. The red arrow highlights Te detection in the spectrum.

The growth of *P. pabuli* ALJ109b in the presence and in the absence of Te (IV) was followed. The strain was able to grow in up to 5 × 10^–4^ M Te (IV). Specific growth rates considered early and late (8 h) exponential growth time points, based on the growth curve for this strain for strain *P. pabuli* ALJ109b. The specific growth rates were similar to the control condition in concentrations up to 2.5 × 10^–4^ M Te (IV) but decreased at the concentration of 5 × 10^–4^ M Te (IV) (Figure 1A). *Escherichia coli* BL21 was not able to grow in the presence of Te (IV).

Considering that 5 × 10^–4^ M Te (IV) was the lowest concentration that affected *P. pabuli* ALJ109b growth, Te (IV) reduction was evaluated at this concentration. At a concentration of 5 × 10^–4^ M Te (IV), *P. pabuli* ALJ109b showed Te (IV) depletion efficiencies in the order of 1.25 Δmg.DO^–1^ and a reduction rate at 8 h of 0.06 Δmg.DO^–1^.h^–1^ (Table 1). This reduction rate allowed for a removal of 20.66% of initial Te (IV) within 8 h, reaching 33.17% in the later stationary phase (20 h).

**TABLE 1:** Reduction efficiencies, reduction rates, and percentage of Te (IV) depletion all through the growth of *Paenibacillus pabuli* ALJ109b in the presence of 5 × 10^–4^ M of Te (IV).

Time (h)	2	4	6	8	20
Re (Δmg.DO^–1^)	28.89	21.71	16.53	4.14	3.19
SD (±)	0.17	0.28	0.18	0.12	0.07
Rr (Δmg.DO^–1^.h^–1^)	14.45	5.43	2.75	0.52	0.16
SD (±)	0.08	0.07	0.03	0.02	0.00
Te (IV) depletion (%)	18.31	17.03	24.70	20.66	33.17
SD (±)	0.86	0.68	0.76	0.76	0.97

A visual demonstration of Te (IV) reduction was observed in SEM imaging of *P. pabuli* ALJ109b with 5 × 10^–4^ M Te (IV). Te-containing nanoparticles are visualized in electron-dense aggregates of structures with clear spheroid organization (Figure 1B). The observed spheroid structures are sized at the nanometer scale, < 100 nm, and therefore can be classified as nanoparticles.

### Metabolic and Stress-Related Impact of Te (IV)

**FIGURE 2:**
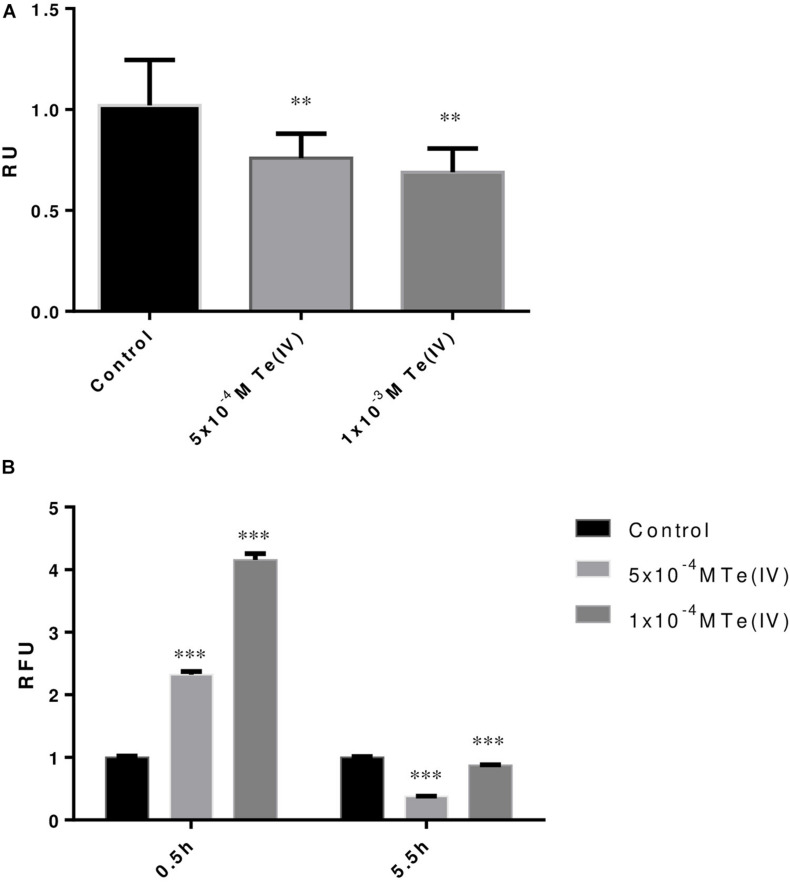
Tellurite-induced stress response of *Paenibacillus pabuli* ALJ109b. **(A)** MTT assay of strain *P. pabuli* ALJ109b. Relative units (RU) represent the ratio between the absorbance (MTT) of each treatment compared to control (without metal). **(B)** ROS assays showing two incubation periods with H_2_DCFDA. Relative fluorescence units (RFU) represent the ratio between the fluorescence intensity (ROS) of each treatment compared to control (without metal). Data shown are the mean values (±standard deviations) obtained from three independent experiments. Significant difference of values from treatment from the value of Control computed by one-way ANOVA ^∗∗^*p* ≤ 0.01, ^∗∗∗^*p* ≤ 0.001.

Variation in metabolic activity in response to Te (IV) was tracked by using MTT assay. MTT assay demonstrated that in the presence of 1 × 10^–3^ M of Te (IV), *P. pabuli* ALJ109b dropped its activity by 17% when compared to the control situation (Figure 2A). The response to oxidative stress induced by Te (IV) was demonstrated by evaluating the production of reactive oxygen species using a ROS assay in *P. pabuli* ALJ109b. ROS formation increased 2.3-fold at the concentration of 5 × 10^–4^ M of Te (IV), when compared to the control without Te (IV). Continuous tracking of ROS formation revealed that *P. pabuli* ALJ109b, when grown in the presence of Te (IV), was able to maintain or even decrease its intracellular ROS levels compared to the control situation after 5 h and 30 min (Figure 2B).

### Genomic and Proteomic Potential for Te (IV) Resistance

Draft genomes of *P. pabuli* ALJ109b were obtained from Illumina sequencing. *P. pabuli* ALJ109b’ had a 6.8-Mb genome, which was assembled into 46 contigs. A total of 6,105 identified CDS regions, 6,210 genes, 7 rRNAs, 1 tmRNA, and 97 tRNAs were identified. Potential occurrence of plasmid analysis, using PlasFlow software package, did not identify any plasmid-marked contig. A genome phylogenetic identification of *Paenibacillus* strain ALJ109b identified the strain as belonging to the species *Paenibacillus pabuli* (*P. pabuli* ALJ109b) with a FastANI score of 99.04% similarity.

A detailed analysis of genetic determinants with relation to Te (transport, resistance, reduction) was performed by PSI-Blast search of the annotated genome. No known Te (IV) transporters were identified in the *P. pabuli* ALJ109b genome. Few genetic determinants with experimentally confirmed Te (IV) resistance activity were detected. These included a near-complete *ars* operon (Pp_CDS_2955 to Pp_CDS_2957), as well as the isolated *ter* operon component, *terC* (Pp_CDS_900), and a *kilA* gene from the *kilAB*/*cysK* gene cluster Pp_CDS_1611. Gene-coding proteins with demonstrated Te (IV)-reducing ability were identified and are further characterized in the last section of the results, *vide infra*.

The comparative proteomic analysis of *P. pabuli* proteins obtained in both Te (IV)-treated and no-treatment conditions was determined using five independent biological replicates. Using the NCBI pipeline annotation from the genome sequence of *P. pabuli* ALJ109b, a reference proteome was created from the strains’ complete CoDing Sequences (CDS). The number of CDS regions detected and identified corresponds to 44% (2828) proteins of its reference proteome. Of the 2,828 proteins, 59% of these annotated sequences (1667) were assigned to, at least, one functional pathway.

### Impact of Te (IV) in Metabolic Pathways

**FIGURE 3:**
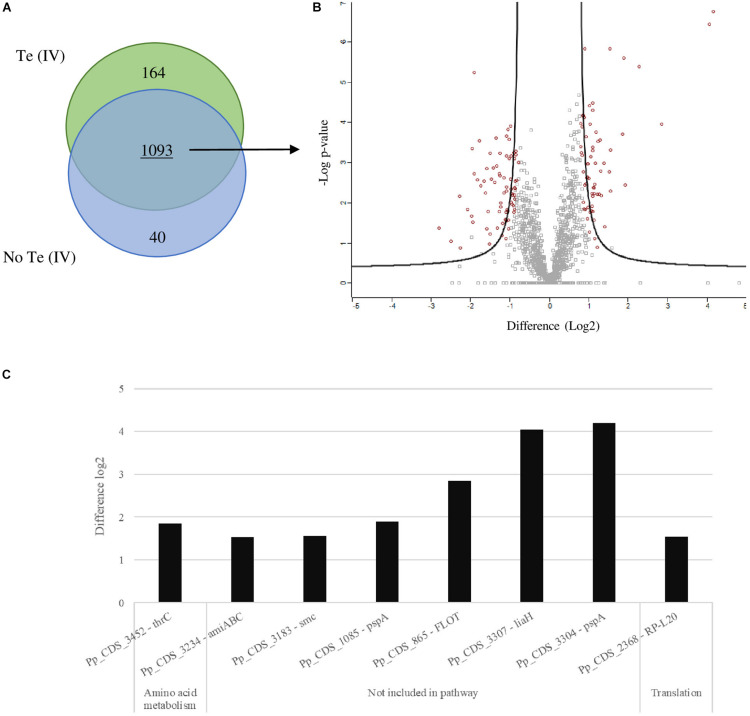
Differential proteomics in *Paenibacillus*. Schematic representations of the determination of significant change in abundance (SCA) of proteins obtained for growth of *Paenibacillus pabuli* ALJ109b in Control [without Te (IV)] and 5 × 10^–4^ M of Te (IV). Each test condition was performed in triplicate. **(A)** Venn diagram representing the total proteins obtained, exclusive to each test and combined. **(B)** Volcano plot showing total shared proteins between tests (Welsh t test; FDR-0.05; S0 > 1), those with SCA indicated by large red circles. **(C)** Highest overexpressed proteins (log2 over 1.5) result from LC-MS analyses; proteins are grouped according to KEGG functional pathways.

The analysis of the proteomes obtained by LC-MS revealed some shifts in the metabolic pathways of cells grown in the presence of Te (IV). The proteome resulting from the growth of *P. pabuli* ALJ109b, with and without Te (IV), reveals 1,832 identifiable proteins. From these, 204 proteins were exclusively found when the strain was grown in one of the conditions (Figure 3A). In more detail, 164 proteins were exclusively found in the presence of Te (IV), 68 had a positive significant change in abundance (SCA), and 75 additional proteins had a negative SCA (Figures 3A,B). In the absence of Te (IV), 40 proteins were exclusively found. A full list of exclusive and SCA proteins can be found in the supplementary material (Supplementary Tables 1, 2).

The detected and identified SCA and exclusive proteins that were assigned to a functional pathway were used to determine the activation or inactivation of the pathways they were a part of. The significance of the activation/inactivation of each pathway was calculated based on the number of proteins detected in relation to the size of the pathway (number of proteins in the pathway present in the reference proteome). The growth of *P. pabuli* ALJ109b in the presence of Te (IV) was associated with a significant change in the representation of the metabolic pathway (level 3) of ABC transporters. When comparing the ABC transporter SCA proteins in both Te (IV) and control growth, we observed that three proteins contribute positively to the metabolic pathway (D-ribose pyranase [EC:5.4.99.62]; L-cystine transport system substrate-binding protein and the potD—spermidine/putrescine transport system substrate-binding protein) and three proteins contributed negatively (LplA, putative aldouronate transport system substrate-binding protein; LplB, putative aldouronate transport system permease protein; and TroB, manganese/zinc/iron transport system ATP- binding protein) (Supplementary Figure 1). The protein Pp_CDS_1334—potD is linked to stress response by the same mechanism described for stress response mediated by lysin described by Olin-Sandoval et al. (2019). Therefore, in *P. pabuli* ALJ109 the polyamine-harvesting mechanism, observed by potD overexpression, may be part of the response to the oxidative stress observed by ROS reduction over time. It is also shown that a detailed analysis of overexpressed proteins, co-located in the genome, highlights other pathways that are overexpressed in *P. pabuli* ALJ109b. Several clusters of amino acids biosynthesized are overexpressed or are exclusive in the presence of Te (IV) such as Pp_CDS_700/701—lysine biosynthesis; Pp_CDS_724/726/730—methionine salvage; Pp_CDS_2315/2316—methionine synthesis; and Pp_CDS_3451–3453—threonine and homoserine synthesis (Supplementary Figure 2). Two other pathways are highlighted, the overexpressed *lia* operon and the Te (IV) exclusive *ars* operon. Other overexpressed pathways remain with unknown function (Supplementary Figure 2).

### Proteins of Interest in Te (IV) Reduction

The presence of Te (IV) induced a significant change in the abundance of proteins that are not assigned to specific pathways, as is the case of thioredoxin reductase (EC 1.8.1.9) involved in defense against oxidative stress. Evaluation of proteins independently shows that the highest overregulation was observed for Pp_CDS_3304—PspA, phage shock protein A; Pp_CDS_3307—LiaH, similar to PspA; and the Pp_CDS_865—FLOT, flotillin, with increases of log_2_ 4.2, log_2_ 4.1, and log_2_ 2.8 times (Figure 3C; Supplementary Table 1), respectively. Apart from the aforementioned, some other proteins are also significantly overexpressed (Figure 3C). These are mostly not included in any functional metabolic pathway, except for Pp_CDS_3452—thrC involved in amino acid metabolism and Pp_CDS_2368—RP-L20, a constituent of the ribosomal machinery. Of those not included in any functional pathway, most are implicated in stress response, such as the abovementioned *lia* operon elements and FLOT; the remaining Pp_CDS_3183—smc is involved in chromosome condensation and partitioning, and Pp_CDS_3234—amiABC is involved in peptidoglycan recycling. A significant number of proteins identified remain hypothetical or with unrecognized function (Supplementary Table 1).

A detailed analysis of the *P. pabuli* ALJ109b genome allowed the identification of proteins with either demonstrated Te (IV)-reducing activity, i.e., nitrate reductase EC 1.7.99.4 (Sabaty et al., 2001), thioredoxin reductase EC 1.8.1.9, alkyl hydroperoxide reductase EC 1.11.1.26 (Arenas-Salinas et al., 2016), dihydrolipoamide dehydrogenase EC 1.8.1.4 (Arenas et al., 2014), Isocitrate dehydrogenase EC 1.1.1.42 (Reinoso et al., 2013) or FAD-dependent oxireductase EC 1.4.3.3 (Pugin et al., 2014) or the putative Te (IV)-reducing activity, i.e., catalase EC 1.11.1.6 (Calderón et al., 2006), 6-phosphogluconate dehydrogenase EC 1.1.1.44 (Sandoval et al., 2015) or Type II—NADH dehydrogenase EC 1.6.99.3 (Díaz-Vásquez et al., 2015). For the proteins with hypothetical Te (IV)-reducing activity, all those with a molybdopterin-containing motif found in the *P. pabuli* ALJ109b genome—oxidoreductase molybdopterin-binding (superfamily) (Pp_CDS_1271); uncharacterized molybdopterin-containing oxireductase YuiH (Pp_CDS_1962), and CTP:molybdopterin cytidylyltransferase EC 2.7.7.76 (Pp_CDS_4487)—were included. All the proteins identified were recovered in the high-throughput proteomic analysis except for mercury reductase (EC 1.16.1.1); flavorubredoxin reductase (EC 1.7.2.5), and the putative pyridine nucleotide-disulfide oxidoreductase YkgC. None of the proteins displayed an SCA in the presence of Te (IV) (Table 2); in the case of flavorubredoxin, this is due to the protein only being required in anaerobioses.

**TABLE 2:** Identification of known proteins with Te (IV)-reducing ability and proteins with putative Te (IV)-reducing ability in the *Paenibacillus pabuli* ALJ109b reference proteome with abundance change (SCA) when strain ALJ109b grows in the presence of 5 × 10^–4^ M of Te (IV).

Protein	Reference proteome ID	log2 difference
Nitrate reductase EC 1.7.99.4	Pp_CDS_1648	No SCA
Thioredoxin reductase EC 1.8.1.9	Pp_CDS_151	No SCA
Alkyl hydroperoxide reductase EC 1.11.1.26	Pp_CDS_2353	No SCA
Flavorubredoxin reductase EC 1.7.2.5	Not found	–
Mercuric reductase EC 1.16.1.1	Not found	–
Putative pyridine nucleotide-disulfide oxidoreductase YkgC	Not found	–
Dihydrolipoamide dehydrogenase EC 1.8.1.4	Pp_CDS_558	No SCA
	Pp_CDS_2587	
	Pp_CDS_4841	
FAD-dependent oxireductase EC 1.4.3.3	Pp_CDS_234	No SCA
Tipe II—NADH dehydrogenase EC 1.6.99.3	Pp_CDS_1274	No SCA
	Pp_CDS_1275	
	Pp_CDS_3377	
	Pp_CDS_3392	
	Pp_CDS_4316	
	Pp_CDS_5529	
Catalase EC 1.11.1.6	Pp_CDS_117	No SCA
	Pp_CDS_197	
	Pp_CDS_1308	
	Pp_CDS_2224	
	Pp_CDS_5110	
	Pp_CDS_5236	
6-Phosphogluconate dehydrogenase EC 1.1.1.44	Pp_CDS_2448	No SCA
	Pp_CDS_3328	
	Pp_CDS_5214	
Isocitrate dehydrogenase EC 1.1.1.42	Pp_CDS_1977	
**Molybdopterin-containing proteins**		
Oxidoreductase molybdopterin-binding (superfamily)	Pp_CDS_1271	No SCA
Uncharacterized molybdopterin-containing oxireductase YuiH	Pp_CDS_1962	
CTP:molybdopterin cytidylyltransferase EC 2.7.7.76	Pp_CDS_4487	

**FIGURE 4:**
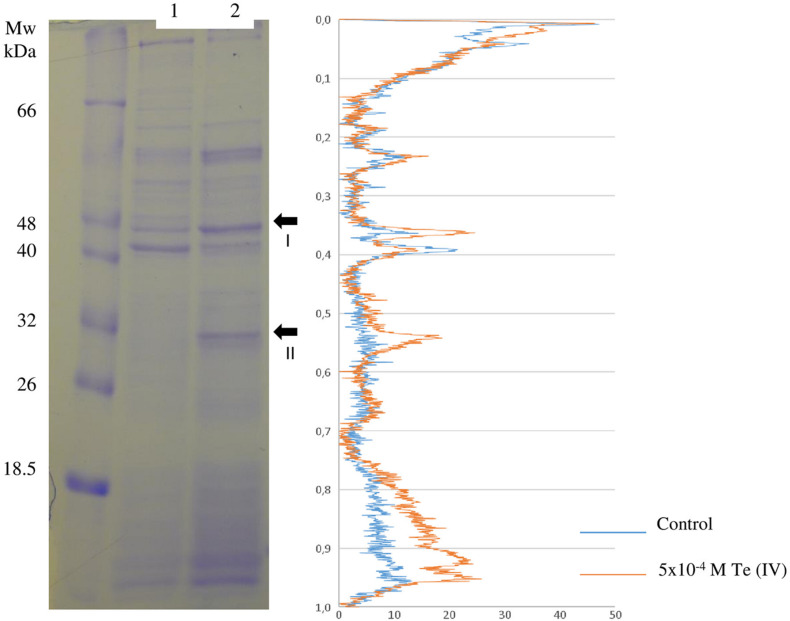
Differential expression of total proteins from *Paenibacillus pabuli* ALJ109b. (Right) Denaturing gel comparing total protein from (1) Control (without metal) and (2) 5 × 10^–4^ M Te (IV). Fragments indicated in lane 2 (arrows) were excised, purified, and identified by MS/MS, with resulting identification in the table. (Left) Lane comparison of band intensities and densitogram, from control (blue) and metal treatment (orange).

In contrast, the protein profile analysis, obtained by SDS–PAGE, revealed two proteins with clear overexpression in the presence of 5 × 10^–4^ M Te (IV), enolase, and flagellin (Figure 4 and Table 3). Viewing the LC-MS results, no enolase or phosphopyruvate hydratase homologue is also found exclusively or overexpressed in the presence of 5 × 10^–4^ M Te (IV). This result is therefore not clear. A deeper understanding of the metal-reducing ability of flagellin was performed.

**TABLE 3:** Identification by MS of fragments purified from denaturing 2D gel electrophoresis (Figure 4).

ID	Description	Coverage (%)	Peptides	AAs	MW (kDa)
I	Enolase OS = Bacillus sp. FJAT-27264 OX = 1850362 GN = eno PE = 3 SV = 1	44	17	428	45.7
II	Flagellin OS = Bacillus filamentosus OX = 1402861 GN = B1B01_04555 PE = 3 SV = 1	5	2	286	31

### Characterization of Te (IV)-Reducing Ability of Flagellin

**FIGURE 5:**
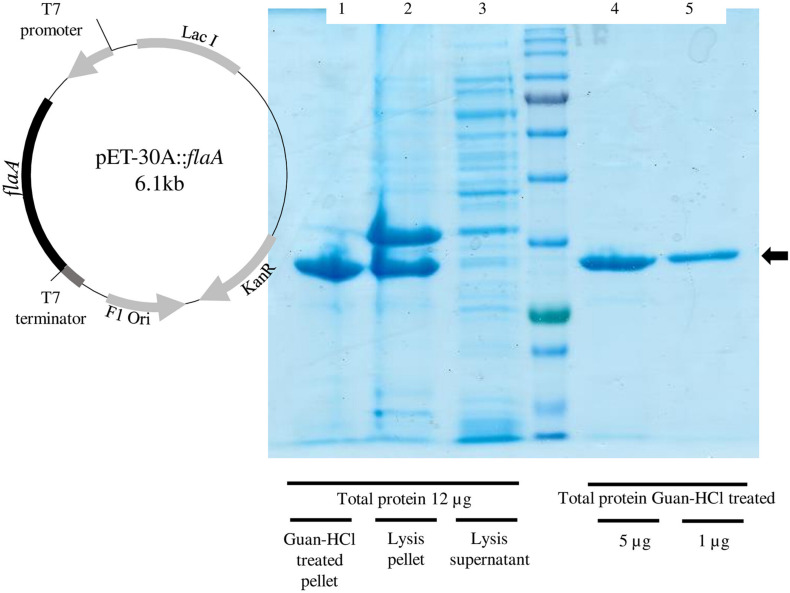
Confirmation of FlaA expression and purification in *Escherichia coli* BL21. (Right) Graphical representation of recombinant plasmid pET-30A::*flaA.* (Left) Denaturing electrophoresis with demonstration of *flaA* expression (black arrow). Lane 1—post lysis guanidine–HCl-treated fraction; lane 2—the post lysis-insoluble fraction; lane 3—post lysis-soluble fraction; all samples loaded are normalized with 12 μg of total protein. In lanes 4 and 5 are, respectively, 5 and 2 μg of total protein, post guanidine–HCl treatment.

**FIGURE 6:**
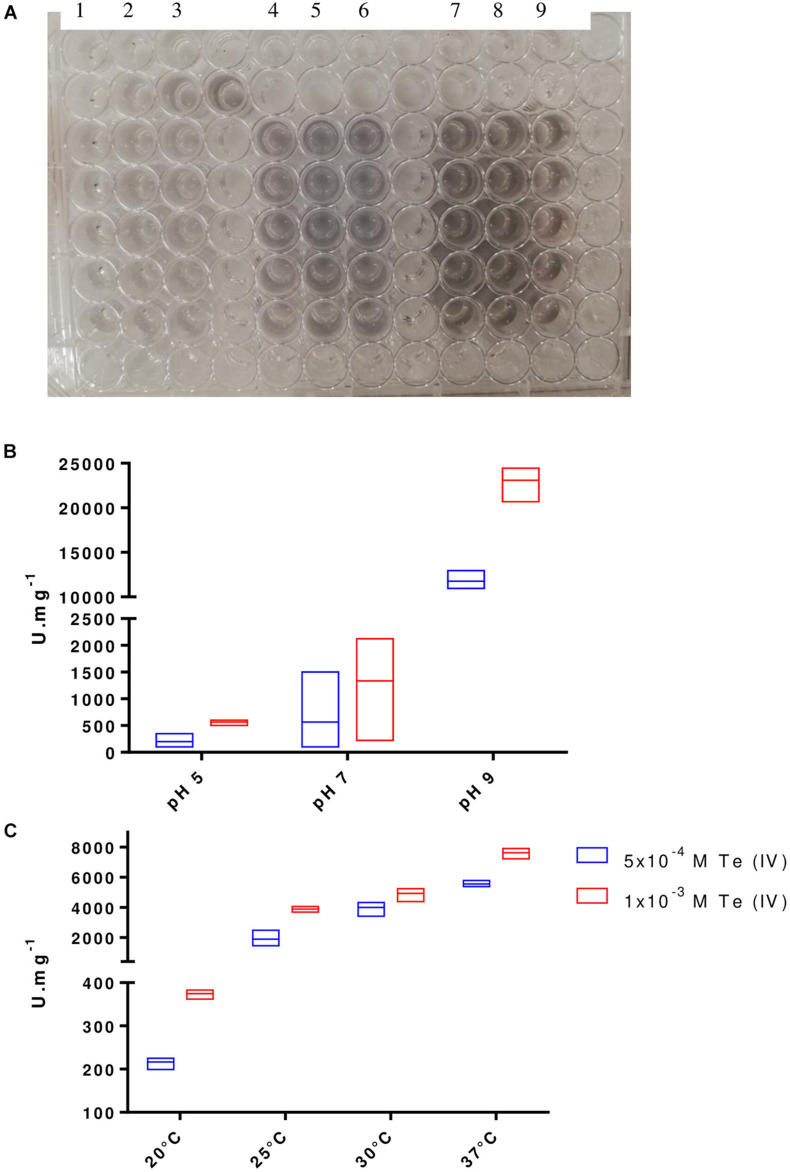
Te (0) formation by FlaA. **(A)** Te (0) formation visible by the appearance of black precipitates in each reaction mixture (individual wells). Test conditions represented Control—columns 1, 2, 3; 5 × 10^–4^ M Te (IV)—columns 4, 5, 6; and 1 × 10^–3^ M Te (IV)—columns 7, 8, 9. Optimal pH **(B)** and temperature **(C)** of Te (0) forming activity for FlaA at 1 μg with 2 Te (IV) concentrations, 5 × 10^–4^ M Te (IV) and 1 × 10^–3^ M Te (IV). Boxes indicate mean and higher/lower value from three independent replicates.

The cloning of *P. pabuli* AL109b flagellin in *E. coli* BL21 produced a 37-kDa protein that was used for Te (IV) reduction (Figure 5). As described by most literature, heterologous flagellin often produced inclusion bodies during protein extraction protocols. This was resolved with an incubation in guanidine HCl that resolubilized the protein (Figure 5). Te (IV)-reducing assays demonstrated that flagellin is effective in reducing Te (IV) to its elemental form Te (0) (Figure 6A). Levels of Te (0) formation were variable depending on pH, temperature, and Te (IV) concentration. Higher pH increased Te (0) formation with a peak activity of 24,450 U.mg^–1^ at pH 9, in 1 × 10^–3^ M Te (IV) (Figure 6B). An increase in temperature was mostly followed by an increase in Te (0) formation with peak reducing activity increasing from 567 to 23,100 U.mg^–1^ from 20°C to 37°C, in 1 × 10^–3^ M Te (IV) (Figure 6C). Results obtained in higher pH and temperature conditions were more reproducible. The rate of Te (0) formation, in most test conditions, increased with the increase in initial Te (IV) concentration from 5 × 10^–4^ to 1 × 10^–3^ M of Te (IV) and reached a plateau at the highest concentration of 2 × 10^–3^ M of Te (IV) (Figures 6A–C).

Analysis of the *P. pabuli* ALJ109b FlaA sequence showed a 256-amino acid protein; it is included in the group of flagellins with the shortest length and therefore one with a short, exposed domain D2/D3 (≈136–169). A comparison with closely related FlaA sequences from *P. pabulis* strains reveals that N and C terminal D0 domains remain conserved with higher sequence variations observed in N terminal D1a/b domains and D2 D3 domains. Overall, FlaA (Pp_CDS_1131) contains a more positive net charge and contains a higher number of long R groups.

## Discussion

The evaluation Te (IV) resistance of *P. pabuli* AL109b and its ability to maintain similar growth kinetics in the presence of Te (IV), up to 2.5 × 10^–4^ M, indicates a highly resistant phenotype, over 1-fold from the best *Paenibacillus* sp. described in the literature (Chien and Han, 2009). Resistance to a concentration of 5 × 10^–4^ M Te (IV) is significantly higher than that reported for strains with known Te (IV) resistance mechanisms such as *Escherichia coli* with *ter*BCDE (Kormutakova et al., 2000), or similar to the resistance demonstrated for *R. capsulatus*, a model system for the study of the microbial interaction with Te (IV) for the last decades (Borghese et al., 2014).

*P. pabuli* ALJ109b reduced 20.7% of Te (IV) in 8 h from a solution at a concentration of 5 × 10^–4^ M. Reporting Te (IV) reduction efficiency and reduction rates this way limits comparison but allows for determination of the efficiency of processes that rely on biomass limitations and have specific timeframes. Nevertheless, the rate of Te (IV) reduction is, to our understanding, high. Still, the analysis of the strain genome showed that the reduction ability was not related to known specific Te (IV) reduction mechanisms. The residue formed by the Te reduced was composed of spherical structures of less than 100 μm, classifying them as nanoparticles. All the structures observed present the same shape, indicating a monodisperse synthesis unlike what is seen for *Bacillus selenireducens* that form nanorods, shards, and rosettes (Baesman et al., 2007) or the membrane fractions of *Lysinibacillus sp.* ZYM-1 that form various shapes of Te plates (Zhang et al., 2010). Instead, the monodisperse synthesis of Te nanostructures by *P. pabuli* ALJ109b resembles that of *Rhodococcus aetherivorans* BCP1 (Presentato et al., 2018) or *Bacillus sp.* BZ (Zare et al., 2012). A monodisperse bioproduction of spheroid-shaped nanoparticles represents a promising new process in nanoparticle production.

The first evaluation of the impact of Te on the metabolism of *P. pabuli* ALJ109b was performed using MTT to follow the activity of the cells, and by quantifying ROS formation. The cells reduced their activity up to 17% in the presence of Te (IV). The presence of Te (IV) induced the formation of ROS, as previously described in other strains (Chasteen et al., 2009). By continuously tracking ROS formation, it was clear that *P. pabuli* ALJ109b activated mechanisms to counteract Te (IV)-induced ROS, justifying looking for the proteins involved in the control of the excess ROS formed. The analysis of the metabolic pathways that are selected in the presence of Te (IV) showed a significant change in the representation of the metabolic pathway (level 3) of ABC transporters supported in PotD overexpression, which was previously linked to the response to the oxidative stress (Olin-Sandoval et al., 2019). It is noteworthy that none of the proteins overexpressed in ABC transporter metabolic function are indicative of a Te (IV) transporter, which validates the previous hypothesis that metal efflux is not a Te (IV) resistance mechanism in this strain (Llyod-Jones et al., 1994).

A detailed analysis of overexpressed proteins, co-located in the genome, highlights that clusters of amino acid biosynthesis are overexpressed in *P. pabuli* ALJ109b. This effect is opposite to what is seen in proteomic studies where, under Ni or Cd stress, bacterial cells decrease amino acid synthesis (Cheng et al., 2009; Izrael-Živković et al., 2018). In the particular case of *P. pabuli* ALJ109b, the biosynthesis of specific amino acids may not be a result of increased protein synthesis, as this pathway is not overrepresented. Instead, the biosynthesis of amino acids may be related to the production of intermediaries in specific pathways. A particular example is lysine harvesting and biosynthesis which has been demonstrated to stimulate NADPH production to prevent imbalances in the redox state under oxidative conditions (Olin-Sandoval et al., 2019). Moreover, the *lia* operon and the Te (IV)-exclusive *ars* operon are also overexpressed. In previous works, the *lia* operon was identified as a genetic mechanism involved in cell envelope stress response (Suntharalingam et al., 2009). Regarding the *ars* operon, it has been proposed that the arsenical efflux pump ArsC is involved in modifying the substrate-binding site of the anion-translocating ATPase, thus conferring moderate levels of resistance to Te (IV) (Turner et al., 1992a).

The presence of Te (IV) induced a significant change in the abundance of proteins that were not assigned to a metabolic pathway (KEGG pathway database), as is the case of some of the proteins with the highest fold change in the presence of Te (IV). The overexpression of PspA and LiaH, phage shock protein A, and flotillin are indicative of stress response activation in *P. pabuli* ALJ109 involving cell wall integrity maintenance under Te (IV) exposure. The protein PspA and its homologue LiaH, is often recognized, in differential proteomic profiling as a marker protein for stress response, acting by maintaining cytoplasmic membrane integrity and/or the proton-motive force (Wenzel et al., 2012; Tsai et al., 2015). These may contribute to preservation of cell wall integrity, essential for the maintenance of cellular metabolic activity demonstrated by the MTT assay. Other proteins that could also be related to the maintenance of the homeostasis of the cell, identified as overexpressed, were related to ribosomal machinery, chromosome condensation and partitioning, and recycling of peptidoglycan (Supplementary Figure 1 and Supplementary Table 1). These proteins neither are known Te (IV) reducers nor present the enzymatic activity commonly associated with Te (IV)-reducing ability, i.e., presence of molybdenum as a cofactor. The genomic and proteomic analysis of the genome revealed the existence of several proteins with demonstrated or putative Te (IV)-reducing activity (Table 1), but none were significantly increased in the presence of Te (IV). No possible comparison could be made with previous reports since there are no reported proteomic studies that allow an inter-genus comparison of Te (IV) reduction. Therefore, an undescribed mechanism must be responsible for Te (IV) reduction in *P. pabuli* ALJ109b.

The 2D denaturing electrophoresis was used also to obtain a differential protein expression, and the results differed from LC-MS analysis. Enolase and flagellin, detected by SDS-PAGE, were not detected overexpressed in LC-MS results. Detection of flagellin may be limited for being bonded to Te. As is the case for metallothioneins, a flagellin-Te molecule may be resistant to the proteolytic activity of trypsin (Wang et al., 2007). Metal binding to exposed amino acid residues impedes the binding of trypsin to lysine and/or arginine residues and its proteolytic activity. Enolase, like the abovementioned proteins, has not an expected enzymatic activity commonly associated with a Te (IV) reducer. On the other hand, the overexpression observed for flagellin may be a direct response to the presence of Te (IV). Other studies already demonstrated the ability of flagellin monomers of binding several metals such as Ag, Au, Cu, Co, Pd, and Cd (Kumara et al., 2007), Pb (Chen et al., 2019), and Ag (Gopinathan et al., 2013), to surface-exposed amino acid residues. Flagellin has been associated with TeNP production in *Rhodobacter capsulatus* (Borghese et al., 2020) without being determined its function, if any, in the TeNP assembly.

The heterologous produced flagellin was used to evaluate the Te (IV) binding to flagellin and reduction to its elemental form Te (0). *P. pabuli* ALJ109 flagellin showed a higher reducing activity at 4°C and for a temperature up to 37°C. When compared to previous results, FlaA demonstrates a Te (0) formation activity, at similar pH and temperature, higher than the flavoproteins from *E. coli* NorW and YkgC, ∼660 and 870 U.mg^–1^ protein, respectively, and lower than *E. coli* flavoprotein GorA, ∼30,000 U.mg^–1^ protein (Arenas-Salinas et al., 2016). When comparing Te (0) formation activity with crude cell extracts from multiple strains (Figueroa et al., 2018), FlaA outperforms all extracts in an average of 10-fold higher activity. To this date, Te (IV) reduction has been reported for several proteins (Table 1), but for most proteins their involvement in further nanoparticle formation has not been described. Further characterization of flagellin from *P. pabuli* ALJ109b could add knowledge in biobased strategies to Te (IV) reduction and/or TeNP formation.

## Conclusion

In this study, we identified a highly Te (IV)-resistant *Paenibacillus* strain from an industrial resulting environment. The genome sequencing analysis and differential proteomics revealed a specific metabolic response to Te (IV) in *P. pabuli* ALJ109b for the first time. The response to Te (IV) involved the overexpression of marker proteins for stress response such as phage shock protein and the chaperon PotD. Proteins related to oxidative stress response, particularly associated with cell wall or cell envelope, are overrepresented. Te (IV) showed to induce ROS generation that the strain solved by activating specific metabolic pathways. The genomic and high-throughput proteomics analyses did not identify any known Te (IV) resistance mechanisms; nevertheless, *P. pabuli* ALJ109b uses Te (IV) reduction as a defense mechanism. We demonstrated that *P. pabuli* ALJ109b uses flagellin, FlaA, as a Te (IV)-reducing agent and that this protein has a high Te (0) formation activity at room temperature and pH of 9.

It is also demonstrated in this work for the first time the metabolic response to Te (IV) in a highly resistant *Paenibacillus* strain. The flagellin purified from *P. pabuli* ALJ109b is an effective Te (IV) reducer with potential in nanoparticle fabrication.

## Methods

### Bacterial Strain Isolation and Growth

*Paenibacillus pabuli* ALJ 109b was isolated from a mine sediment originated in the Aljustrel copper mine (37°52′07.3″N 8°09′24.7″W), in southern Portugal. Sediment samples were suspended in 50% diluted LB. The samples were incubated at 25°C for 7 days in an orbital shaker. The culture medium was incremented with sodium tellurite (Sigma-Aldrich, St. Louis, MO, United States) at regular times, increasing from 5 × 10^–4^, 1 × 10^–3^, 3 × 10^–3^, 5 × 10^–3^, up to 1 × 10^–2^ M. Prior to each Te (IV) enrichment, an aliquot of the suspension was plated in 50% diluted LB agar for selection of isolates.

For Te (IV) resistance assay, two strains were tested, *Escherichia coli* BL21 (commercially obtained) and *P. pabuli* ALJ109b. *E. coli* BL21 was tested for growth in Te (IV) to demonstrate if the strain was resistant to Te (IV). Blackening of the growth media was indicative of Te (IV) reduction. Both were tested in LB with increasing concentrations of Te (IV), 1 × 10^–4^, 2.5 × 10^–4^, and 5 × 10^–4^ M, while comparing against growth in the absence of Te (IV). *E. coli* BL21 was incubated at 37°C wile *P. pabuli* LJ109b at 25°C. Statistically significant variations of the specific growth rates, for each Te (IV) concentration versus control, were determined by performing a t test, using GraphPad Prism version 8.0, ^∗^ *p* ≤ 0.05.

### Tellurite Reduction and Nanoparticle Formation in *P. pabuli* ALJ109b

The reduction of Te (IV) by *P. pabuli* ALJ109b was determined at 5 × 10^–4^ M. Aliquots for Te (IV) reduction testing were recovered at four times, lag/early exponential, mid exponential, late exponential, and late stationary growth phases. Cells were centrifuged 20 min at 4000 *g*, the pellets were preserved for further tests, and the supernatant was stored for evaluation of Te (IV) reduction. Quantitative depletion of sodium Te (IV) was quantified using a chromophore diethyldithiocarbamate (DDTC) method adapted from Turner and colleagues (Turner et al., 1992b). The reagent mixture was prepared with final concentrations of 1 mM DDTC and 0.5 M Tris–HCl pH 7 buffer, and each sample was incubated for no more than 15 min prior to absorbance reading at 340 nm. Quantitative data were obtained from a minimum of three experimental replicates.

The efficiency of Te (IV) depletion (reduction efficiency—Re) was determined as the ratio of the absolute variation of Te (IV) in grams, from time 0 (T0) to late exponential growth (Tf), per growth, expressed as a variation on optical density, Tf – T0. The reduction rate was determined as reason of the Re per time at Tf, as demonstrated in the equation.

$$R_{e}=\frac{\left|\Delta \,T\,e\right|}{\Delta \,D\,O\,\left(\mathrm{Tf}-\mathrm{To}\right)}\;R_{r}=R_{e}\,/\,t\,\left(T\,f\right)$$

Demonstration of Te precipitation was performed by scanning electron microscopy with coupled energy-dispersive X-ray spectroscopy (SEM-EDS), in backscattered electrons mode (BSE). The evaluation was made on cell preparations recovered from the late exponential phase in the presence of 5 × 10^–4^ M Te (IV). Cell pellets from cultures were collected by centrifugation at 4000 *g*, washed twice in saline phosphate buffer (PBS 1×), and resuspended in 0.1 ml of the same buffer.

Droplets of cell concentrate ≈30 μl were dried in a 5 × 5-mm stainless steel plate, at room temperature, followed by two-step fixation with 2.5% glutaraldehyde and by dehydration with increasing ethanol concentration, 70%/80%/90%/95%.

SEM micrographs were obtained on a FEI Quanta 400 FEG ESEM, and EDS analysis was accomplished using an Oxford INCA Energy 350 equipped with the SAMX IDEFIX software, with an accelerating voltage of 15 kV and a beam current of 20 nÅ.

### Tellurite-Induced Stress Response

Stress response was determined by tracking the regulation of metabolic activity using 3-(4,5-dimethylthiazol-2-yl)-2,5-diphenyltetrazolium bromide assay (MTT assay) (Caldeira et al., 2020) and by determining the formation of reactive oxidative species (ROS) by using 2,7-dichlorofluorescein-diacetate (H_2_DCFDA) assay (Jakubowski, 2000). Cells were grown in LB broth supplemented with Te (IV), 5 × 10^–4^ M, 1 × 10^–3^ M, and a control without metal. For MTT assays, strain *P. pabuli* ALJ109b was incubated for 6 h, collected by centrifugation at 13.300 *g* for 10 min, and washed twice with growth media. Dilutions were prepared to obtain cell suspensions with OD 0.2 in growth media. For formazan crystal formation, 200 μl of cell suspension was mixed with 20 μl of MTT solution and incubated for 1 h at 25°C. Crystals were retrieved by centrifugation at 13.300 *g* for 2 min; these were then resuspended in 2.5 ml of DMSO and incubated 1 h at room temperature. Absorbance of the mixture solution was read at 550 nm. For ROS determination, the strain was incubated until reaching an OD of 0.3. Cells were washed twice with PBS, incubated in 25 μM H_2_DCFDA for 30 min at 25°C, retrieved by centrifugation, and again washed twice with PBS. Intracellular ROS levels were determined by lysing cell pellets by pasteurization, for 20 min at 80°C. After centrifugation, supernatants were collected and fluorescence were read hourly during 15 h (λem = 527 nm and λex = 495 nm). For both MTT and ROS assays, the values were compared as the ratio between the values of the test condition (with metal) and the value of the control without metal. All assays were performed in triplicates. Statistical significance accessed by one-way ANOVA between test conditions replicates means, using GraphPad Prism version 8.0, ^∗∗^*p* ≤ 0.01, ^∗∗∗^*p* ≤ 0.001.

### Genome Sequencing, Annotation, and Strain Identification

Strain AlJ109b was grown in liquid media LB broth, streaked from a single colony. Cells were collected, and DNA was extracted using a DNeasy PowerSoil Kit (Qiagen), according to manufacturer instructions. Libraries of total genomic DNA were prepared using Nextera XT Preparation Kit (Illumina, San Diego, CA, United States) following the manufacturer’s instructions. Libraries were purified using HighPrep PCR Clean-up beads (MagBio Genomics, Inc.). Fragment analyzer 5200 (Agilent NGS Fragment 1-6000 pb methods) was used to check the fragment size distribution and molarity of each library. Nine-picomolar libraries were sequenced on an Illumina MiSeq System based at the Section of Microbiology in the Department of Biology of Copenhagen University with 2 × 300bp chemistry (MiSeq Reagent Kit v3). Pairing, trimming, and assembly based on Bruijn graphs were performed using CLC Genomics Workbench v9.5.4 (Qiagen) using default parameters. Resulting contigs were submitted to GhostKOALA (KEGG Orthology And Links Annotation) annotated genomes as reference proteome (Kanehisa et al., 2016). In GhostKOALA, Kegg identifiers (K numbers) were assigned to the sequence data by GHOSTX searches, against a nonredundant set of KEGG GENES. Genome annotation was performed upon submission to the GenBank databank using NCBI Prokaryotic Genome Annotation Pipeline for determination of coding sequences (CDS) as well as RNA sequences. Potential occurrence of plasmids was determined by searching for genomic signatures using PlasFlow 1.1 software package (Krawczyk et al., 2018).

Genome phylogeny was determined by using rMLST (Jolley et al., 2012) and PhyloPhlan (Segata et al., 2013) analyses, and similarity results were calculated by average nucleotide identity, using ANI calculator, Kostas software (Goris et al., 2007).

### Comparative Methodologies for Differential Proteomics

For determining the impact of Te (IV) in total protein expression, *P. pabuli* ALJ109b was grown in LB broth containing Te (IV), 5 × 10^–4^ M, 1 × 10^–3^ M, or a control without metal. Upon reaching the late exponential growth phase, cells were collected by centrifugation and washed twice in PBS 1×.

For the comparison of differential proteomics using denaturing gel electrophoresis, the cell pellet was resuspended in 0.9 ml STB solution (0.075 g.l^–1^ Tris, 0.345 ml.l^–1^ HCl (1.72 N), 0.5 ml.l^–1^ β-mercaptoethanol, and 0.5 g.l^–1^ sacarose) and mixed after adding 0.1 ml of SDS 20%. Cell suspension was sonicated with continued on/off cycles of 10 s for 4 min, on an ice water mixture, heated at 95°C for 10 min, and cooled on ice. Lastly, the suspension was centrifuged at 14,000 rpm for 10 min, and the supernatant was harvested. Total protein obtained was quantified by using Bradford reagent (Bio-Rad^®^, Hercules, CA, United States), and 12 μg of total protein was aliquoted by mixing with 7 μl of loading buffer (Morris formulation) and boiled 10 min before loading on a denaturing gel. Protein separation was obtained in a 12% acrylamide/bisacrylamide denaturing gel (SDS 0.1%). Electrophoresis was performed at room temperature for 1 h at 120 V. The molecular marker used for size reference (kDa) was the Low Molecular Weight Protein Marker (NZYTech, Lisboa, Portugal). Visualization of proteins was performed by staining with Coomassie Blue followed by destaining with a methanol/acetic acid solution. From the visual analysis and densitogram comparison (Quantity One, Bio-Rad), selected fragments were excised and stored in ultrapure water for MS/MS identification.

For the comparison of differential proteomics using LC-MS, cell pellets from treated and untreated conditions were lysed by resuspension in lysis buffer (guanidinium hydrochloride 6 M, tris(2-carboxyethyl)phosphine (TCEP) 10 mM, 2-chloroacetamide (CAA) 40 mM, HEPES 50 mM, pH 8.5). Samples were heated and disrupted by sonication as mentioned above and normalized at 30 μg for trypsin digestion. The samples were four-fold diluted in digestion buffer (acetonitrile (ACN) 10%, HEPES 50 mM pH 8.5) and then incubated for 4 h with trypsin (1:100 trypsin-to-protein ratio) (Sigma T6567) at room temperature with horizontal shaking at 500 rpm. Trypsin was inactivated with trifluoroacetic acid, and debris was removed by centrifugation (10,000 *g*, 10 min). The tryptic peptides were fractionated using a stage tip protocol as described by Rappsilber (Rappsilber et al., 2007). A total of three C18 plugs were gently punched out from the filter disk with the help of the sampling tool syringe. Plugs were placed at the tip of a 200-μl pipette tip with a plunger and activated with 30 μl methanol by centrifugation at 1,000 *g* for 2 min, followed by 30 μl 100% ACN, and finally 2 × 30 μl of 3% ACN with 1% TFA. Peptides were loaded onto the filter unit by centrifugation at 1,000 *g*. Bound peptides were washed twice using 30 μl of 0.1% formic acid (FA). Peptides were eluted using two rounds of 30 μl 60% ACN in 0.1% FA, with centrifugation between each round. Liquid was evaporated, and peptides were redissolved in 2% ACN with 1% TFA. The peptide concentration in the samples was estimated with a NanoDrop, and 1.5 μg peptide was loaded for analysis on a Q Exactive (Thermo Scientific, Bremen, Germany).

### Mass Spectrometry

The samples were analyzed by liquid chromatography tandem mass spectrometry (LC-MS/MS), and data were recorded in a data-dependent manner, automatically switching between MS and MS/MS acquisition, on a Q Exactive (Thermo Scientific, Bremen, Germany). An EASY nLC-1000 liquid chromatography system (Thermo Scientific, Odense, Denmark) was coupled to the mass spectrometer through an EASY-Spray source, and peptide separation was performed on 15-cm EASY-Spray columns (Thermo Scientific) with 2-μm-size C18 particles and the inner diameter of 75 μm. The mobile phase consisted of solvents A (0.1% FA) and B (80% ACN in 0.1% FA). The initial concentration of solvent B was 6%, and hereafter gradients were applied to reach the following concentrations: 14% B in 18.5 min, 25% B in 19 min, 38% B in 11.5 min, 60% B in 10 min, 95% B in 3 min, and 95% B for 7 min. The total length of the gradient was 70 min. The full scans were acquired in the Orbitrap with a resolution of 120,000, and a maximum injection time of 50 ms was applied. For the full scans, the range was adjusted to 350–1,500 m/z. The top 10 most abundant ions from the full scan were sequentially selected for fragmentation with an isolation window of 1.6 m/z (Kelstrup et al., 2012) and excluded from re-selection for a 60-s time period. For the MS/MS scans, the resolution was adjusted to 120,000 and maximum injection time of 80 ms. Ions were fragmented in a higher-energy collision dissociation cell with normalized collision energy of 32% and analyzed in the Orbitrap.

### Construction and Purification of a Recombinant *P. pabuli* ALJ109b Flagellin

With information provided by the genome of *P. pabuli* ALJ109b, a set of cloning primers was designed for the insertion of the *flaA* gene in plasmid pET 30A, *Eco*RI_flaA (sense) 5′ CCG GAA TTC ATG ATT ATC AAT CAC AAC TTA CCA, and *Sal*I_flaA_R (antisense) 5′ ACG GCG TCG ACT TAA CGA AGC AAG GAC AA. Amplification of the target sequence was performed using the abovementioned primers in a PCR reaction, for a final volume of 50 μl, using 2 U Platinum^TM^ Taq DNA Polymerase (Invitrogen), 0.2 mM of each dNTP, PCR Buffer (1×), 1.5 mM MgCl_2_, 0.4 μM primers, and 2 ng DNA template. The PCR program involved initial denaturation at 94°C (5 min), followed by 30 cycles of 94°C (1 min), 61°C (1 min), and 72°C (45 s).

The PCR-amplified DNA fragments with approximately 700 bp, as well as the plasmid pET 30A, were digested with the restriction enzymes *Eco*RI and *Sal*I. The digested amplified fragments were purified and ligated into the pET 30A expression vector for 1 h at room temperature using 0.5 U of T4 DNA ligase (Thermo Scientific, Waltham, MA, United States). The resulting plasmid pET 30A::flaA was transformed into competent *E. coli* BL21 cells. The correct construction was confirmed by sequencing the complete DNA fragments cloned into the plasmid (Stabvida). *E. coli* BL21 bacterial cells, containing the plasmid pET 30A::flaA, were grown in LB broth containing kanamycin (50 μg.ml^–1^), at 37°C 140 rpm. Inducing agent IPTG (Sigma-Aldrich) was added (5 × 10^–4^ M) at an optical density of 0.5 (Abs 600 nm), and cells resumed growth for 5 h. Cells were harvested by centrifugation at 4,000 *g* for 15 min, resuspended in protein lysis buffer STB, and lysed by mechanical sheering in an Emulsiflex^®^-C3 High-Pressure Homogenizer (Avestin, ONCE, Canada), 2 cycles at 1,500–2,000 psi. The lysis product was centrifuged 10,000 *g*, for 20 min, the supernatant harvested and stored, and the resulting pellet subjected to a guanidine–HCl (6 M) treatment for 1 h at 30°C. Finally, a soluble fraction was obtained by centrifugation at 10,000 *g*, for 20 min, aliquoted, and stored at 4°C in the presence of a proteinase inhibitor complete, EDTA-Free (Roche, Basel, Switzerland). Confirmation of the recombinant protein FlaA was performed in a denaturing gel electrophoresis as described above using as a size (kDa) reference the NZYColour Protein Marker II (NZYTech).

### Demonstration of *in vitro* Te (IV) Reduction Ability by FlaA

Demonstration of the Te (IV)-reducing ability by FlaA was determined by incubating the protein extract with increasing concentrations of soluble Te (IV) and tracking the formation of elemental Te spectrophotometrically, by measuring the absorbance at 500 nm. Protocol was adapted from Figueroa and colleagues (Figueroa et al., 2018). All tests were performed in a final volume of 200 μl with 1 μg of FlaA, in a buffer mixture containing Tris–HCl pH 8, 50 mM, K_2_H_2_PO_4_/KHPO_4_ (1:1) 50 mM, and β-mercaptoethanol 1 mM. Determination of optimal Te (IV)-reducing activity by FlaA was tested with variations in initial Te (IV) concentration from 0 M (control) to 5 × 10^–5^ M to 1 × 10^–3^ M (5 × 10^–4^ M increments), variation in pH from 5, 7, to 9, and variation in temperature from 20°C, 25°C, 30°C, to 37°C. Results are expressed in units of Te (0) formation activity (U) with U = 1 equivalent to an increase of 0.001 in absorbance (500 nm) per minute per volume of reaction. Specific activity was calculated as U per mg of protein. All tests were conducted in triplicates.

## Data Availability Statement

The datasets presented in this study can be found in online repositories. The names of the repository/repositories and accession number(s) can be found below: http://www.proteomexchange.org/, PXD017546; https://www.ncbi.nlm.nih. gov/genbank/, PRJNA606039.

## Author Contributions

PF did the data curation, performed all benchwork, analyzed all data using bioinformatic and statistical analyses and wrote the original draft. RF did the heading of field sampling and processing of some analyses in the laboratory, assisting in strain isolations and growth and reduction assays, reviewed the statistical analyses, and reviewed and edited the manuscript. LM headed in genome sequencing and analyses of sequencing data and reviewed and edited the manuscript. JH headed the proteomic assays and analyses of data resulting from proteomic analyses and reviewed and edited the manuscript. AP performed all imaging assays included in the form of scattering electronic microscopy micrographs. SS conceptualized part of the experiment, supervised the laboratory and bioinformatic analyses on the genome sequencing and proteomics, and reviewed and edited the manuscript. PVM conceptualized the whole experiment and secured the funding, supervised the laboratory, bioinformatics, and statistical analyses, and contributed to the original draft and revised the manuscript. All authors contributed to the article and approved the submitted version.

## Conflict of Interest

The authors declare that the research was conducted in the absence of any commercial or financial relationships that could be construed as a potential conflict of interest.

## Publisher’s Note

All claims expressed in this article are solely those of the authors and do not necessarily represent those of their affiliated organizations, or those of the publisher, the editors and the reviewers. Any product that may be evaluated in this article, or claim that may be made by its manufacturer, is not guaranteed or endorsed by the publisher.

## Supplementary Material

Supplementary Figure 1 Pathways from *Paenibacillus pabuli* ALJ109b showing metabolic change in the presence of Te (IV). SCA proteins were mapped with subsystems classifications from KEGG, top extended bar plot with positive SCA and bottom extended bar plot with negative SCA. Level 3 KEGG pathways were analyzed for regulation using a Fisher’s exact test. FDR adjusted *p*-values are presented for each pathway, *p*-values equal or under 0.05 were considered for determining significant pathways. Black bars display pathway size compared to the size of reference proteome, which can be grouped in pathways. Blue bars display the ratio of SCA proteins in the pathway compared to the total amount of SCA proteins in pathways.

Supplementary Figure 2 Integration of proteomic information in genome *Paenibacillus pabuli* ALJ109b. Schematic representation of over/down expressed and exclusive identified protein positioned in the theoretical arrangement of the genome (merged contigs). Highlighted features of, contiguous, over expressed pathways are detailed table.
