# Supplementary figures and images for: Impact of Tellurite on the Metabolism of Paenibacillus pabuli AL109b With Flagellin Production Explaining High Reduction Capacity
Source: Front Microbiol. 2021 Sep 7;12:718963. doi: 10.3389/fmicb.2021.718963 (PMC8453073; doi:10.3389/fmicb.2021.718963)

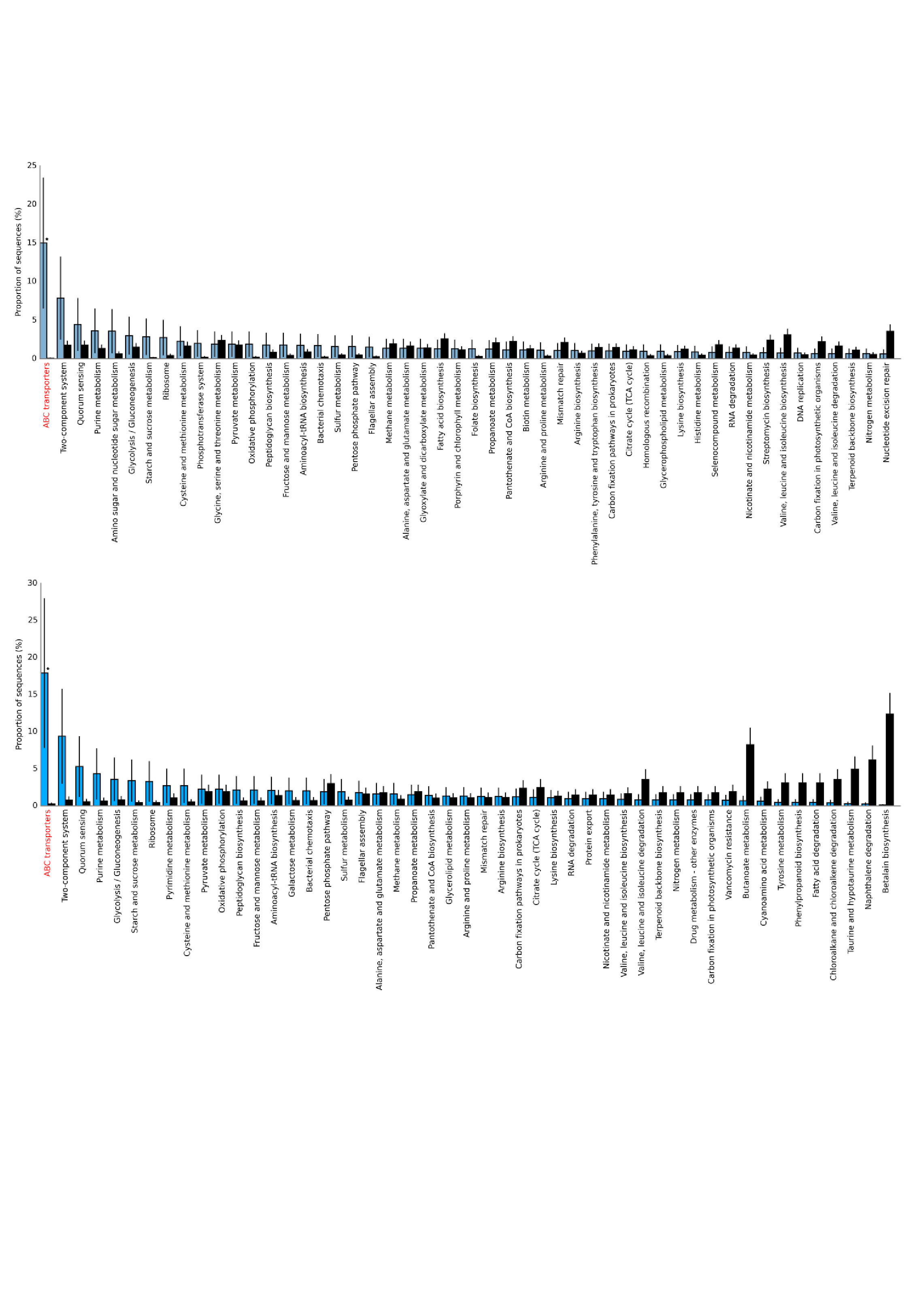

Supplement: Supplementary Figure 1 — Pathways from Paenibacillus pabuli ALJ109b showing metabolic change in the presence of Te (IV). SCA proteins were mapped with subsystems classifications from KEGG, top extended bar plot with positive SCA and bottom extended bar plot with negative SCA. Level 3 KEGG pathways were analyzed for regulation using a Fisher’s exact test. FDR adjusted p-values are presented for each pathway, p-values equal or under 0.05 were considered for determining significant pathways. Black bars display pathway size compared to the size of reference proteome, which can be grouped in pathways. Blue bars display the ratio of SCA proteins in the pathway compared to the total amount of SCA proteins in pathways. [file Image_1.JPEG]

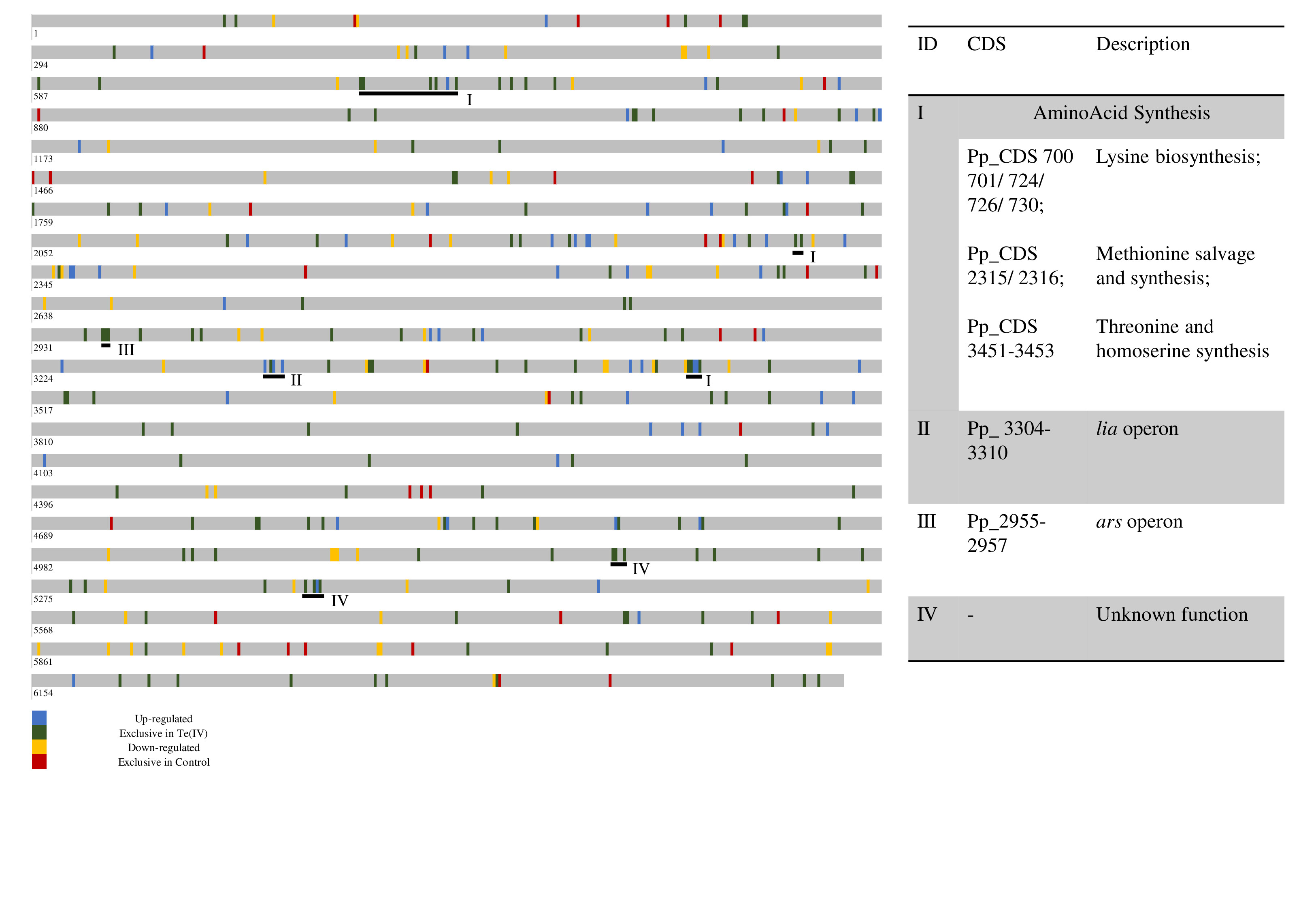

Supplement: Supplementary Figure 2 — Integration of proteomic information in genome Paenibacillus pabuli ALJ109b. Schematic representation of over/down expressed and exclusive identified protein positioned in the theoretical arrangement of the genome (merged contigs). Highlighted features of, contiguous, over expressed pathways are detailed table. [file Image_2.JPEG]

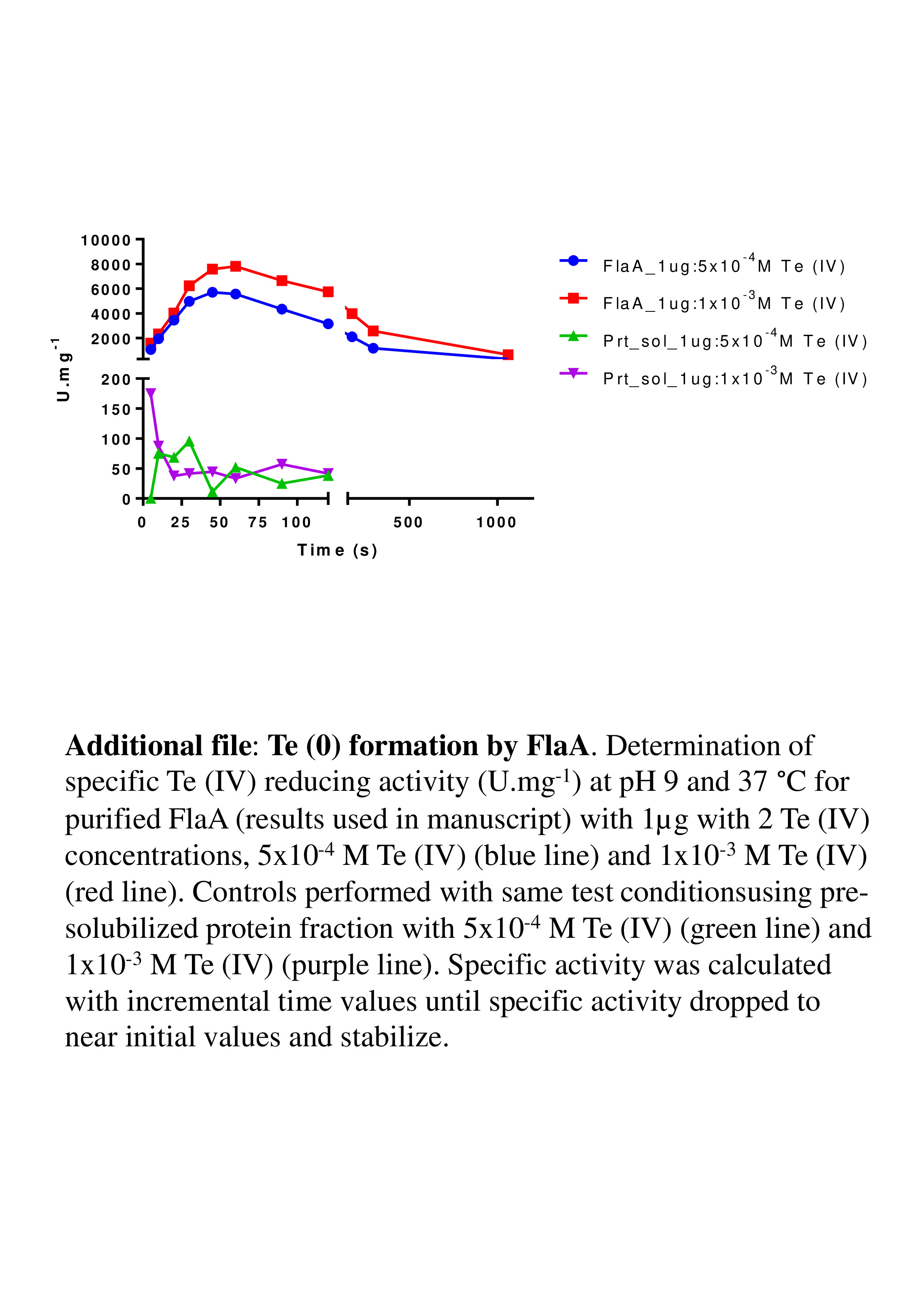

Supplement: Supplementary file 3 [file Image_3.JPEG]
