# Supplementary material for: Impact of Tellurite on the Metabolism of Paenibacillus pabuli AL109b With Flagellin Production Explaining High Reduction Capacity
Source: Front Microbiol. 2021 Sep 7;12:718963. doi: 10.3389/fmicb.2021.718963 (PMC8453073; doi:10.3389/fmicb.2021.718963)
Supplement: Supplementary file 4 [file Data_Sheet_1.PDF]

Study on the impact of tellurite on the metabolism of *Paenibacillus pabuli* AL109b with flagellin production explaining high reduction capacity

Supplementary Table 1. *Paenibacillus pabuli* ALJ109b annotated CDS with significant variation between tested conditions

PRT-ID	KO	Definition	Difference log_2 (welsh t-test)	Significance (q - welsh t-test)
**Pp_CDS_5086**	K00656	E2.3.1.54; pflD; formate C-acetyltransferase [EC:2.3.1.54]	-2.8	0.007
**Pp_CDS_5085**	K04069	pflA; pyruvate formate lyase activating enzyme [EC:1.97.1.4]	-2.5	0.001
**Pp_CDS_773**	K03839	fldA; flavodoxin I	-2.3	0.007
**Pp_CDS_5087**	K04072	adhE; acetaldehyde dehydrogenase / alcohol dehydrogenase [EC:1.2.1.10 1.1.1.1]	-2.3	0.021
**Pp_CDS_3694**	K00363	nirD; nitrite reductase (NADH) small subunit [EC:1.7.1.15]	-2.0	0.003
**Pp_CDS_518**	K15777	DOPA; 4,5-DOPA dioxygenase extradiol [EC:1.13.11.-]	-1.9	0.000
**Pp_CDS_6135**	K07069	uncharacterized protein	-1.9	0.005
**Pp_CDS_3066**	K06416	spoVS; stage V sporulation protein S	-1.7	0.004
**Pp_CDS_3359**	K11710	troB; manganese/zinc/iron transport system ATP- binding protein	-1.7	0.000
**Pp_CDS_3422**	K00240	sdhB; succinate dehydrogenase / fumarate reductase, iron-sulfur subunit [EC:1.3.5.1 1.3.5.4]	-1.6	0.001
**Pp_CDS_5895**	K00294	E1.2.1.88; 1-pyrroline-5-carboxylate dehydrogenase [EC:1.2.1.88]	-1.5	0.030
**Pp_CDS_5008**	K11991	tadA; tRNA(adenine34) deaminase [EC:3.5.4.33]	-1.5	0.001
**Pp_CDS_5365**	K01489	cdd; cytidine deaminase [EC:3.5.4.5]	-1.4	0.001
**Pp_CDS_2290**	K06284	abrB; transcriptional pleiotropic regulator of transition state genes	-1.3	0.008
**Pp_CDS_3449**	K00826	E2.6.1.42; branched-chain amino acid aminotransferase [EC:2.6.1.42]	-1.3	0.034
**Pp_CDS_1444**	K09807	uncharacterized protein	-1.3	0.006
**Pp_CDS_457**	K02874	RP-L14; large subunit ribosomal protein L14	-1.2	0.027
**Pp_CDS_2665**	K00027	ME2; malate dehydrogenase (oxaloacetate-decarboxylating) [EC:1.1.1.38]	-1.2	0.034
**Pp_CDS_692**	K03886	MQCRA; menaquinol-cytochrome c reductase iron-sulfur subunit [EC:1.10.2.-]	-1.1	0.035
**Pp_CDS_5887**	K00259	ald; alanine dehydrogenase [EC:1.4.1.1]	-1.1	0.047
**Pp_CDS_852**	K00874	kdgK; 2-dehydro-3-deoxygluconokinase [EC:2.7.1.45]	-1.1	0.035
**Pp_CDS_2642**	K01807	rpiA; ribose 5-phosphate isomerase A [EC:5.3.1.6]	-1.1	0.023
**Pp_CDS_1546**	K00020	mmsB; 3-hydroxyisobutyrate dehydrogenase [EC:1.1.1.31]	-1.1	0.026
**Pp_CDS_4459**	K01619	deoC; deoxyribose-phosphate aldolase [EC:4.1.2.4]	-1.1	0.031
**Pp_CDS_2581**	K01104	E3.1.3.48; protein-tyrosine phosphatase [EC:3.1.3.48]	-1.0	0.025
**Pp_CDS_2253**	K00860	cysC; adenylylsulfate kinase [EC:2.7.1.25]	-1.0	0.041
**Pp_CDS_4456**	K03784	deoD; purine-nucleoside phosphorylase [EC:2.4.2.1]	-1.0	0.027
**Pp_CDS_113**	K00661	maa; maltose O-acetyltransferase [EC:2.3.1.79]	-1.0	0.030
**Pp_CDS_423**	K01770	ispF; 2-C-methyl-D-erythritol 2,4-cyclodiphosphate synthase [EC:4.6.1.12]	-1.0	0.031
**Pp_CDS_3464**	K03610	minC; septum site-determining protein MinC	-1.0	0.039
**Pp_CDS_3421**	K00239	sdhA; succinate dehydrogenase / fumarate reductase, flavoprotein subunit [EC:1.3.5.1 1.3.5.4]	-1.0	0.039
**Pp_CDS_1143**	K02406	fliC; flagellin	-1.0	0.036
**Pp_CDS_3002**	K07095	uncharacterized protein	-0.9	0.047
**Pp_CDS_4829**	K03711	fur; Fur family transcriptional regulator, ferric uptake regulator	-0.9	0.040
**Pp_CDS_1630**	K00005	gldA; glycerol dehydrogenase [EC:1.1.1.6]	-0.9	0.043
**Pp_CDS_5300**	K05810	yfiH; polyphenol oxidase [EC:1.10.3.-]	-0.9	0.046
**Pp_CDS_2088**	K00850	pfkA; 6-phosphofructokinase 1 [EC:2.7.1.11]	-0.9	0.045
**Pp_CDS_1624**	K03829	yedL; putative acetyltransferase [EC:2.3.1.-]	-0.9	0.038
**Pp_CDS_5600**	K03624	greA; transcription elongation factor GreA	-0.9	0.038
**Pp_CDS_4863**	K03602	xseB; exodeoxyribonuclease VII small subunit [EC:3.1.11.6]	-0.8	0.046
**Pp_CDS_3123**	K03411	cheD; chemotaxis protein CheD [EC:3.5.1.44]	-0.8	0.050
**Pp_CDS_444**	K02358	tuf; elongation factor Tu	0.8	0.046
**Pp_CDS_436**	K02935	RP-L7; large subunit ribosomal protein L7/L12	0.8	0.046
**Pp_CDS_3800**	K00384	trxB; thioredoxin reductase (NADPH) [EC:1.8.1.9]	0.8	0.043
**Pp_CDS_5777**	K02990	RP-S6; small subunit ribosomal protein S6	0.8	0.047
**Pp_CDS_2160**	K00973	E2.7.7.24; glucose-1-phosphate thymidylyltransferase [EC:2.7.7.24]	0.8	0.038
**Pp_CDS_1971**	K01962	accA; acetyl-CoA carboxylase carboxyl transferase subunit alpha [EC:6.4.1.2 2.1.3.15]	0.9	0.036

**Pp_CDS_2358**	K01653	E2.2.1.6S; acetolactate synthase I/III small subunit [EC:2.2.1.6]	0.9	0.047
**Pp_CDS_2596**	K00648	fabH; 3-oxoacyl-[acyl-carrier-protein] synthase III [EC:2.3.1.180]	0.9	0.036
**Pp_CDS_1189**	K02113	ATPF1D; F-type H+-transporting ATPase subunit delta	0.9	0.040
**Pp_CDS_3086**	K12574	rnj; ribonuclease J [EC:3.1.-.-]	0.9	0.048
**Pp_CDS_819**	K22132	tcdA; tRNA threonylcarbamoyladenosine dehydratase	0.9	0.035
**Pp_CDS_3071**	K03553	recA; recombination protein RecA	0.9	0.032
**Pp_CDS_178**	K00133	asd; aspartate-semialdehyde dehydrogenase [EC:1.2.1.11]	0.9	0.040
**Pp_CDS_2243**	K00899	mtnK; 5-methylthioribose kinase [EC:2.7.1.100]	0.9	0.044
**Pp_CDS_2526**	K03665	hflX; GTPase	0.9	0.043
**Pp_CDS_3068**	K18682	rny; ribonucrease Y [EC:3.1.-.-]	0.9	0.033
**Pp_CDS_1993**	K00859	coaE; dephospho-CoA kinase [EC:2.7.1.24]	1.0	0.035
**Pp_CDS_730**	K08969	mtnE; aminotransferase [EC:2.6.1.-]	1.0	0.039
**Pp_CDS_2231**	K21142	moaX; MoaE-MoaD fusion protein [EC:2.8.1.12]	1.0	0.033
**Pp_CDS_3584**	K01595	ppc; phosphoenolpyruvate carboxylase [EC:4.1.1.31]	1.0	0.039
**Pp_CDS_2550**	K08967	mtnD; 1,2-dihydroxy-3-keto-5-methylthiopentene dioxygenase [EC:1.13.11.53 1.13.11.54]	1.0	0.034
**Pp_CDS_6168**	K02035	ABC.PE.S; peptide/nickel transport system substrate-binding protein	1.0	0.046
**Pp_CDS_4107**	K01092	E3.1.3.25; myo-inositol-1(or 4)-monophosphatase [EC:3.1.3.25]	1.0	0.025
**Pp_CDS_4890**	K01756	purB; adenylosuccinate lyase [EC:4.3.2.2]	1.0	0.027
**Pp_CDS_4919**	K01756	purB; adenylosuccinate lyase [EC:4.3.2.2]	1.0	0.027
**Pp_CDS_335**	K01226	treC; trehalose-6-phosphate hydrolase [EC:3.2.1.93]	1.1	0.035
**Pp_CDS_2019**	K02768	PTS-Fru-EIIA; PTS system, fructose-specific IIA component [EC:2.7.1.202]	1.1	0.032
**Pp_CDS_1895**	K01265	map; methionyl aminopeptidase [EC:3.4.11.18]	1.1	0.033
**Pp_CDS_4034**	K07571	S1 RNA binding domain protein	1.1	0.021
**Pp_CDS_1733**	K07258	dacC; serine-type D-Ala-D-Ala carboxypeptidase (penicillin-binding protein 5/6) [EC:3.4.16.4]	1.1	0.031
**Pp_CDS_4832**	K01515	nudF; ADP-ribose pyrophosphatase [EC:3.6.1.13]	1.1	0.020
**Pp_CDS_2294**	K03856	AROA2; 3-deoxy-7-phosphoheptulonate synthase [EC:2.5.1.54]	1.1	0.023
**Pp_CDS_4023**	K07533	prsA; foldase protein PrsA [EC:5.2.1.8]	1.1	0.027
**Pp_CDS_2305**	K01868	TARS; threonyl-tRNA synthetase [EC:6.1.1.3]	1.1	0.027
**Pp_CDS_1805**	K03816	xpt; xanthine phosphoribosyltransferase [EC:2.4.2.22]	1.1	0.028
**Pp_CDS_4794**	K07335	bmpA; basic membrane protein A and related proteins	1.1	0.034
**Pp_CDS_2359**	K01652	E2.2.1.6L; acetolactate synthase I/II/III large subunit [EC:2.2.1.6]	1.2	0.025
**Pp_CDS_2239**	K09013	sufC; Fe-S cluster assembly ATP-binding protein	1.2	0.007
**Pp_CDS_1172**	K02372	fabZ; 3-hydroxyacyl-[acyl-carrier-protein] dehydratase [EC:4.2.1.59]	1.2	0.022
**Pp_CDS_4040**	K03798	ftsH; cell division protease FtsH [EC:3.4.24.-]	1.2	0.043
**Pp_CDS_3310**	K11618	liaR; two-component system, NarL family, response regulator LiaR	1.3	0.003
**Pp_CDS_3453**	K00003	hom; homoserine dehydrogenase [EC:1.1.1.3]	1.3	0.007
**Pp_CDS_2244**	K08963	mtnA; methylthioribose-1-phosphate isomerase [EC:5.3.1.23]	1.3	0.020
**Pp_CDS_5470**	K01989	ABC.X4.S; putative ABC transport system substrate-binding protein	1.4	0.000
**Pp_CDS_3234**	K01448	amiABC; N-acetylmuramoyl-L-alanine amidase [EC:3.5.1.28]	1.5	0.003
**Pp_CDS_2368**	K02887	RP-L20; large subunit ribosomal protein L20	1.5	0.005
**Pp_CDS_3183**	K03529	smc; chromosome segregation protein	1.6	0.001
**Pp_CDS_3452**	K01733	thrC; threonine synthase [EC:4.2.3.1]	1.9	0.005
**Pp_CDS_1085**	K03969	pspA; phage shock protein A	1.9	0.000
**Pp_CDS_865**	K07192	FLOT; flotillin	2.8	0.000
**Pp_CDS_3307**	K03969	liaH; similar to phage shock protein A	4.1	0.000
**Pp_CDS_3304**	K03969	pspA; phage shock protein A	4.2	0.000

Additional 48 unidentified and/or unassigned (KO) proteins have significant change in abundance, 14 with over regulation in the presence of Te(IV) and 34 down regulated in the presence of Te(IV)

Supplementary Table 2. *Paenibacillus pabuli* ALJ109b annotated CDS exclusive in either tested conditions

ID	KO	Definition	Test
**Pp_CDS_112**	K00661	maa; maltose O-acetyltransferase [EC:2.3.1.79]	Control
**Pp_CDS_1466**	K00765	hisG; ATP phosphoribosyltransferase [EC:2.4.2.17]	Control
**Pp_CDS_1472**	K06023	hprK; HPr kinase/phosphorylase [EC:2.7.11.- 2.7.4.-]	Control
**Pp_CDS_2284**	K17318	lplA; putative aldouronate transport system substrate-binding protein	Control
**Pp_CDS_2612**	K10188	lacE; lactose/L-arabinose transport system substrate-binding protein	Control
**Pp_CDS_3168**	K03470	rnhB; ribonuclease HII [EC:3.1.26.4]	Control
**Pp_CDS_3180**	K09787	uncharacterized protein	Control
**Pp_CDS_3360**	K11708	troC; manganese/zinc/iron transport system permease protein	Control
**Pp_CDS_353**	K13954	yiaY; alcohol dehydrogenase [EC:1.1.1.1]	Control
**Pp_CDS_3695**	K00362	nirB; nitrite reductase (NADH) large subunit [EC:1.7.1.15]	Control
**Pp_CDS_5750**	K04771	degP; serine protease Do [EC:3.4.21.107]	Control
**Pp_CDS_5825**	K10542	mglA; methyl-galactoside transport system ATP-binding protein [EC:3.6.3.17]	Control
**Pp_CDS_5955**	K01737	queD; 6-pyruvoyltetrahydropterin/6-carboxytetrahydropterin synthase [EC:4.2.3.12 4.1.2.50]	Control
**Pp_CDS_6102**	K07171	mazF; mRNA interferase MazF [EC:3.1.-.-]	Control
**Pp_CDS_860**	K01784	galE; UDP-glucose 4-epimerase [EC:5.1.3.2]	Control
**Pp_CDS_1124**	K00145	argC; N-acetyl-gamma-glutamyl-phosphate reductase [EC:1.2.1.38]	Te(IV) $5x10^{-4}$
**Pp_CDS_1304**	K06911	uncharacterized protein	Te(IV) $5x10^{-4}$
**Pp_CDS_1334**	K11069	potD; spermidine/putrescine transport system substrate-binding protein	Te(IV) $5x10^{-4}$
**Pp_CDS_1448**	K06958	rapZ; RNase adapter protein RapZ	Te(IV) $5x10^{-4}$
**Pp_CDS_1460**	K11755	hisIE; phosphoribosyl-ATP pyrophosphohydrolase / phosphoribosyl-AMP cyclohydrolase [EC:3.6.1.31 3.5.4.19]	Te(IV) $5x10^{-4}$
**Pp_CDS_1612**	K07114	yfbK; Ca-activated chloride channel homolog	Te(IV) $5x10^{-4}$
**Pp_CDS_1723**	K03790	rimJ; [ribosomal protein S5]-alanine N-acetyltransferase [EC:2.3.1.267]	Te(IV) $5x10^{-4}$
**Pp_CDS_1748**	K01534	zntA; Cd2+/Zn2+-exporting ATPase [EC:3.6.3.3 3.6.3.5]	Te(IV) $5x10^{-4}$
**Pp_CDS_1749**	K21903	cadC; ArsR family transcriptional regulator, lead/cadmium/zinc/bismuth-responsive transcriptional repressor	Te(IV) $5x10^{-4}$
**Pp_CDS_1785**	K01990	ABC-2.A; ABC-2 type transport system ATP-binding protein	Te(IV) $5x10^{-4}$
**Pp_CDS_1796**	K02275	coxB; cytochrome c oxidase subunit II [EC:1.9.3.1]	Te(IV) $5x10^{-4}$
**Pp_CDS_2018**	K00882	fruK; 1-phosphofructokinase [EC:2.7.1.56]	Te(IV) $5x10^{-4}$
**Pp_CDS_2045**	K01776	murI; glutamate racemase [EC:5.1.1.3]	Te(IV) $5x10^{-4}$
**Pp_CDS_2119**	K06183	rsuA; 16S rRNA pseudouridine516 synthase [EC:5.4.99.19]	Te(IV) $5x10^{-4}$
**Pp_CDS_2150**	K01972	E6.5.1.2; DNA ligase (NAD+) [EC:6.5.1.2]	Te(IV) $5x10^{-4}$
**Pp_CDS_2220**	K01652	E2.2.1.6L; acetolactate synthase I/II/III large subunit [EC:2.2.1.6]	Te(IV) $5x10^{-4}$
**Pp_CDS_2237**	K11717	sufS; cysteine desulfurase / selenocysteine lyase [EC:2.8.1.7 4.4.1.16]	Te(IV) $5x10^{-4}$
**Pp_CDS_226**	K00381	cysI; sulfite reductase (NADPH) hemoprotein beta-component [EC:1.8.1.2]	Te(IV) $5x10^{-4}$
**Pp_CDS_2299**	K11618	liaR; two-component system, NarL family, response regulator LiaR	Te(IV) $5x10^{-4}$
**Pp_CDS_2315**	K01760	metC; cysteine-S-conjugate beta-lyase [EC:4.4.1.13]	Te(IV) $5x10^{-4}$
**Pp_CDS_2317**	K00651	metA; homoserine O-succinyltransferase/O-acetyltransferase [EC:2.3.1.46 2.3.1.31]	Te(IV) $5x10^{-4}$
**Pp_CDS_2354**	K00052	leuB; 3-isopropylmalate dehydrogenase [EC:1.1.1.85]	Te(IV) $5x10^{-4}$
**Pp_CDS_246**	K19689	ampS; aminopeptidase [EC:3.4.11.-]	Te(IV) $5x10^{-4}$
**Pp_CDS_247**	K01895	ACSS; acetyl-CoA synthetase [EC:6.2.1.1]	Te(IV) $5x10^{-4}$
**Pp_CDS_2602**	K07177	Lon-like protease	Te(IV) $5x10^{-4}$
**Pp_CDS_2604**	K00954	E2.7.7.3A; pantetheine-phosphate adenylyltransferase [EC:2.7.7.3]	Te(IV) $5x10^{-4}$
**Pp_CDS_2632**	K06994	putative drug exporter of the RND superfamily	Te(IV) $5x10^{-4}$

**Pp_CDS_2842**	K02529	lacI; LacI family transcriptional regulator	Te(IV) $5x10^{-4}$
**Pp_CDS_2844**	K06726	rbsD; D-ribose pyranase [EC:5.4.99.62]	Te(IV) $5x10^{-4}$
**Pp_CDS_2949**	K03484	scrR; LacI family transcriptional regulator, sucrose operon repressor	Te(IV) $5x10^{-4}$
**Pp_CDS_2957**	K03892	arsR; ArsR family transcriptional regulator, arsenate/arsenite/antimonite-responsive transcriptional repressor	Te(IV) $5x10^{-4}$
**Pp_CDS_2986**	K01834	PGAM; 2,3-bisphosphoglycerate-dependent phosphoglycerate mutase [EC:5.4.2.11]	Te(IV) $5x10^{-4}$
**Pp_CDS_3058**	K00763	pncB; nicotinate phosphoribosyltransferase [EC:6.3.4.21]	Te(IV) $5x10^{-4}$
**Pp_CDS_3083**	K03466	ftsK; DNA segregation ATPase FtsK/SpoIIIE, S-DNA-T family	Te(IV) $5x10^{-4}$
**Pp_CDS_3120**	K09749	uncharacterized protein	Te(IV) $5x10^{-4}$
**Pp_CDS_3149**	K02410	fliG; flagellar motor switch protein FliG	Te(IV) $5x10^{-4}$
**Pp_CDS_3155**	K01419	hslV; ATP-dependent HslUV protease, peptidase subunit HslV [EC:3.4.25.2]	Te(IV) $5x10^{-4}$
**Pp_CDS_322**	K07816	E2.7.6.5X; putative GTP pyrophosphokinase [EC:2.7.6.5]	Te(IV) $5x10^{-4}$
**Pp_CDS_3394**	K00435	hemQ; Fe-coproporphyrin III decarboxylase [EC:1.11.1.-]	Te(IV) $5x10^{-4}$
**Pp_CDS_3411**	K11144	dnaI; primosomal protein DnaI	Te(IV) $5x10^{-4}$
**Pp_CDS_3439**	K01613	psd; phosphatidylserine decarboxylase [EC:4.1.1.65]	Te(IV) $5x10^{-4}$
**Pp_CDS_3450**	K04518	pheA2; prephenate dehydratase [EC:4.2.1.51]	Te(IV) $5x10^{-4}$
**Pp_CDS_3451**	K00872	thrB1; homoserine kinase [EC:2.7.1.39]	Te(IV) $5x10^{-4}$
**Pp_CDS_3454**	K06209	pheB; chorismate mutase [EC:5.4.99.5]	Te(IV) $5x10^{-4}$
**Pp_CDS_3478**	K01698	hemB; porphobilinogen synthase [EC:4.2.1.24]	Te(IV) $5x10^{-4}$
**Pp_CDS_3528**	K01443	nagA; N-acetylglucosamine-6-phosphate deacetylase [EC:3.5.1.25]	Te(IV) $5x10^{-4}$
**Pp_CDS_3529**	K02564	nagB; glucosamine-6-phosphate deaminase [EC:3.5.99.6]	Te(IV) $5x10^{-4}$
**Pp_CDS_3538**	K01990	ABC-2.A; ABC-2 type transport system ATP-binding protein	Te(IV) $5x10^{-4}$
**Pp_CDS_3703**	K03924	moxR; MoxR-like ATPase [EC:3.6.3.-]	Te(IV) $5x10^{-4}$
**Pp_CDS_3706**	K07015	uncharacterized protein	Te(IV) $5x10^{-4}$
**Pp_CDS_3848**	K02016	ABC.FEV.S; iron complex transport system substrate-binding protein	Te(IV) $5x10^{-4}$
**Pp_CDS_3858**	K00262	E1.4.1.4; glutamate dehydrogenase (NADP+) [EC:1.4.1.4]	Te(IV) $5x10^{-4}$
**Pp_CDS_3905**	K01759	GLO1; lactoylglutathione lyase [EC:4.4.1.5]	Te(IV) $5x10^{-4}$
**Pp_CDS_4079**	K18979	queG; epoxyqueuosine reductase [EC:1.17.99.6]	Te(IV) $5x10^{-4}$
**Pp_CDS_4154**	K02424	fliY; L-cystine transport system substrate-binding protein	Te(IV) $5x10^{-4}$
**Pp_CDS_4219**	K02503	HINT1; histidine triad (HIT) family protein	Te(IV) $5x10^{-4}$
**Pp_CDS_426**	K01883	CARS; cysteinyl-tRNA synthetase [EC:6.1.1.16]	Te(IV) $5x10^{-4}$
**Pp_CDS_4504**	K19689	ampS; aminopeptidase [EC:3.4.11.-]	Te(IV) $5x10^{-4}$
**Pp_CDS_4551**	K06878	tRNA-binding protein	Te(IV) $5x10^{-4}$
**Pp_CDS_4766**	K09705	uncharacterized protein	Te(IV) $5x10^{-4}$
**Pp_CDS_4841**	K00382	DLD; dihydrolipoamide dehydrogenase [EC:1.8.1.4]	Te(IV) $5x10^{-4}$
**Pp_CDS_4849**	K21567	fnr; ferredoxin/flavodoxin---NADP+ reductase [EC:1.18.1.2 1.19.1.1]	Te(IV) $5x10^{-4}$
**Pp_CDS_4862**	K13789	GGPS; geranylgeranyl diphosphate synthase, type II [EC:2.5.1.1 2.5.1.10 2.5.1.29]	Te(IV) $5x10^{-4}$
**Pp_CDS_4891**	K01589	purK; 5-(carboxyamino)imidazole ribonucleotide synthase [EC:6.3.4.18]	Te(IV) $5x10^{-4}$
**Pp_CDS_4912**	K03705	hrcA; heat-inducible transcriptional repressor	Te(IV) $5x10^{-4}$
**Pp_CDS_4920**	K01589	purK; 5-(carboxyamino)imidazole ribonucleotide synthase [EC:6.3.4.18]	Te(IV) $5x10^{-4}$
**Pp_CDS_5037**	K06377	spo0M; sporulation-control protein	Te(IV) $5x10^{-4}$
**Pp_CDS_5045**	K16216	yueD; benzil reductase ((S)-benzoin forming) [EC:1.1.1.320]	Te(IV) $5x10^{-4}$
**Pp_CDS_5161**	K12132	prkC; eukaryotic-like serine/threonine-protein kinase [EC:2.7.11.1]	Te(IV) $5x10^{-4}$
**Pp_CDS_5211**	K00800	aroA; 3-phosphoshikimate 1-carboxyvinyltransferase [EC:2.5.1.19]	Te(IV) $5x10^{-4}$
**Pp_CDS_5217**	K01585	speA; arginine decarboxylase [EC:4.1.1.19]	Te(IV) $5x10^{-4}$
**Pp_CDS_5253**	K06972	PITRM1; presequence protease [EC:3.4.24.-]	Te(IV) $5x10^{-4}$
**Pp_CDS_5268**	K02027	ABC.MS.S; multiple sugar transport system substrate-binding protein	Te(IV) $5x10^{-4}$
**Pp_CDS_5288**	K06180	rluD; 23S rRNA pseudouridine1911/1915/1917 synthase [EC:5.4.99.23]	Te(IV) $5x10^{-4}$
**Pp_CDS_5293**	K01870	IARS; isoleucyl-tRNA synthetase [EC:6.1.1.5]	Te(IV) $5x10^{-4}$
**Pp_CDS_5369**	K06217	phoH; phosphate starvation-inducible protein PhoH and related proteins	Te(IV) $5x10^{-4}$

**Pp_CDS_5374**	K07403	nfeD; membrane-bound serine protease (ClpP class)	Te(IV) $5x10^{-4}$
**Pp_CDS_5439**	K00851	E2.7.1.12; gluconokinase [EC:2.7.1.12]	Te(IV) $5x10^{-4}$
**Pp_CDS_5607**	K04780	dhbF; nonribosomal peptide synthetase DhbF	Te(IV) $5x10^{-4}$
**Pp_CDS_5714**	K00662	aacC; aminoglycoside 3-N-acetyltransferase [EC:2.3.1.81]	Te(IV) $5x10^{-4}$
**Pp_CDS_5773**	K02031	ABC.PE.A; peptide/nickel transport system ATP-binding protein	Te(IV) $5x10^{-4}$
**Pp_CDS_5799**	K02313	dnaA; chromosomal replication initiator protein	Te(IV) $5x10^{-4}$
**Pp_CDS_5816**	K03530	hupB; DNA-binding protein HU-beta	Te(IV) $5x10^{-4}$
**Pp_CDS_6011**	K01421	yhgE; putative membrane protein	Te(IV) $5x10^{-4}$
**Pp_CDS_6059**	K06158	ABCF3; ATP-binding cassette, subfamily F, member 3	Te(IV) $5x10^{-4}$
**Pp_CDS_6184**	K03800	lplA; lipoate---protein ligase [EC:6.3.1.20]	Te(IV) $5x10^{-4}$
**Pp_CDS_6194**	K00768	E2.4.2.21; nicotinate-nucleotide--dimethylbenzimidazole phosphoribosyltransferase [EC:2.4.2.21]	Te(IV) $5x10^{-4}$
**Pp_CDS_6425**	K07032	uncharacterized protein	Te(IV) $5x10^{-4}$
**Pp_CDS_700**	K00215	dapB; 4-hydroxy-tetrahydrodipicolinate reductase [EC:1.17.1.8]	Te(IV) $5x10^{-4}$
**Pp_CDS_701**	K01734	mgsA; methylglyoxal synthase [EC:4.2.3.3]	Te(IV) $5x10^{-4}$
**Pp_CDS_71**	K09702	uncharacterized protein	Te(IV) $5x10^{-4}$
**Pp_CDS_724**	K08964	mtnB; methylthioribulose-1-phosphate dehydratase [EC:4.2.1.109]	Te(IV) $5x10^{-4}$
**Pp_CDS_726**	K08965	mtnW; 2,3-diketo-5-methylthiopentyl-1-phosphate enolase [EC:5.3.2.5]	Te(IV) $5x10^{-4}$
**Pp_CDS_733**	K13566	NIT2; omega-amidase [EC:3.5.1.3]	Te(IV) $5x10^{-4}$
**Pp_CDS_767**	K06180	rluD; 23S rRNA pseudouridine1911/1915/1917 synthase [EC:5.4.99.23]	Te(IV) $5x10^{-4}$
**Pp_CDS_823**	K03657	uvrD; DNA helicase II / ATP-dependent DNA helicase PcrA [EC:3.6.4.12]	Te(IV) $5x10^{-4}$
**Pp_CDS_998**	K00134	GAPDH; glyceraldehyde 3-phosphate dehydrogenase [EC:1.2.1.12]	Te(IV) $5x10^{-4}$
